# Pharmacist Intervention for Safer Prescribing in Patients With Type 2 Diabetes at High Risk: A Randomized Clinical Trial

**DOI:** 10.1001/jamanetworkopen.2025.59946

**Published:** 2026-02-18

**Authors:** Lisa K. Gilliam, Melissa M. Parker, Minnie W. Chen, Andrew J. Karter, Richard W. Grant

**Affiliations:** 1 The Permanente Medical Group, South San Francisco Kaiser Medical Center, South San Francisco, California; 2 Kaiser Permanente Northern California Division of Research, Pleasanton

## Abstract

**Importance:**

Severe hypoglycemia is a life-threatening, iatrogenic complication of diabetes medications associated with increased risks of falls, cardiovascular events, cognitive decline, and mortality.

**Objective:**

To determine whether proactive outreach by a clinical pharmacist applying an evidence-based hypoglycemia-prevention algorithm (Hypoglycemia on a Page) would result in safer prescribing of diabetes regimens among patients with type 2 diabetes (T2D) with hypoglycemia risk.

**Design, Setting, and Participants:**

This randomized clinical trial was conducted between July 20, 2023, and January 22, 2024, with follow-up outcomes collected through January 2025. The study included adults with T2D at high risk of hypoglycemia based on a validated hypoglycemia risk tool. The trial was conducted within Kaiser Permanente Northern California, a large, integrated health care delivery system.

**Intervention:**

Patients were randomized 1:1 to either a protocol-driven outreach by a clinical pharmacist (intervention) arm or a usual care (control) arm.

**Main Outcome and Measure:**

The main outcome was the comparison of the proportion of patients who were prescribed safer (less hypoglycemia-prone) diabetes regimens, defined as discontinuation of sulfonylureas and/or rapid-acting or short-acting or mixed insulins, using an intention-to-treat analysis.

**Results:**

From an eligible population of 1204 patients, 200 were enrolled into the clinical trial. Among these, 191 patients were in the intention-to-treat cohort (mean [SD] age, 71.3 [11.5] years; 100 females [52.4%]). The 2 study arms were similar at baseline in terms of race and ethnicity, sex, mean (SD) hemoglobin A_1c_ (HbA_1c_) level (intervention: 8.0 [1.3]; control: 8.3 [1.8]), and number (percentage) of patients receiving insulin (intervention: 82 [85.4%]; control:85 [89.5%]) and sulfonylurea (intervention: 25 [26.0%]; control: 21 [22.1%]). At 6 months, patients in the intervention arm were more likely to be prescribed a safer diabetes regimen than those in the control arm (27 [28.1%] vs 15 [15.8%]; risk difference [RD], 12.3% [95% CI, 0.6% to 24.0%]). Patients in the intervention arm also experienced significantly fewer hypoglycemia-related emergency department or inpatient encounters (0 vs 5 [5.3%]; RD, −5.3% [95% CI, −11.8% to −1.3%]). There was no worsening of HbA_1c_ control in the intervention arm compared with the control arm (47 [61.8%] vs 49 [63.6%] with HbA_1c_ <8%; RD, −1.8% [95% CI, −17.0% to 13.5%]).

**Conclusions and Relevance:**

In this randomized clinical trial, adding proactive, protocol-driven clinical pharmacist outreach into collaborative team-based care improved medication optimization and patient outcomes. This approach provides an evidence-based strategy for reducing the risk of developing severe hypoglycemia in individuals with T2D at high risk, enhancing patient safety, and potentially reducing overall health care cost.

**Trial Registration:**

ClinicalTrials.gov Identifier: NCT06746714

## Key Points

**Question:**

Does proactive outreach by a clinical pharmacist applying an evidence-based hypoglycemia-prevention algorithm result in safer diabetes regimens among patients with type 2 diabetes at high risk of hypoglycemia?

**Findings:**

In this randomized clinical trial of 200 adults, patients in the intervention arm (proactive outreach by the pharmacist) were statistically significantly more likely to be prescribed a safer diabetes regimen compared with those in the usual care arm (28% vs 16%).

**Meaning:**

The findings of this study suggest that proactive, protocol-driven outreach by clinical pharmacists as part of collaborative team-based care leads to safer diabetes medication prescribing in patients with type 2 diabetes at high risk of hypoglycemia.

## Teaser

This randomized clinical trial assesses whether proactive outreach by a clinical pharmacist applying an evidence-based hypoglycemia-prevention algorithm results in safer prescribing of diabetes regimens among patients with type 2 diabetes at high risk of hypoglycemia.

## Introduction

Severe hypoglycemia is a common and potentially life-threatening complication of type 2 diabetes (T2D) treatment. It is associated with increased risks of falls,^1,2^ cardiovascular events,^3,4,5^ cognitive decline,^6^ and mortality,^7,8^ and it contributes substantially to health care utilization and cost.^9,10^ Despite its clinical importance, hypoglycemia risk is often underrecognized and undertreated in routine care,^11^ partly because severe hypoglycemia is most frequently treated outside of the medical system, and traditional glycemic targets emphasize hemoglobin A_1c_ (HbA_1c_) control rather than minimizing glucose variability or preventing iatrogenic hypoglycemia.

Multiple studies have shown that deintensification of hypoglycemia-prone regimens, particularly sulfonylureas and insulin, can reduce hypoglycemia risk without compromising glycemic control.^12^ The International Geriatric Diabetes Society recently convened a Deprescribing Consensus Initiative to provide guidance on how to deprescribe hypoglycemia-prone diabetes medications in older adults^13^; however, systematic approaches to identifying and managing patients at high risk for hypoglycemia are lacking.

Prior interventions in this area have focused on clincian^14^ or patient education^15^ or both.^16^ To our knowledge, there have been no published studies of proactive, guideline-based, and pharmacist-led interventions to reduce diabetes medication risk. To address this gap, we developed Hypoglycemia on a Page (HOAP), an evidence-based, hypoglycemia-prevention algorithm to support safer diabetes medication management, using a Delphi consensus process.^17^ The HOAP algorithm provides guidance on medication regimen changes, glycemic target resetting, use of continuous glucose monitoring, glucagon prescribing, tailoring patient education, and adding hypoglycemia to the patient’s problem list. We then conducted a randomized clinical trial to evaluate whether proactive clinical pharmacist outreach, guided by the HOAP algorithm, would increase the proportion of patients with high risk prescribed safer diabetes regimens compared with usual care. We hypothesized that this intervention would result in more frequent deprescribing of hypoglycemia-prone agents without worsening glycemic control and would reduce hypoglycemia-related emergency department (ED) and inpatient (IP) utilization.

## Methods

The rationale and design for this randomized clinical trial have been described previously.^17^ Our clinical trial was conducted from July 20, 2023, to January 22, 2024, with follow-up outcomes collected through January 2025 within Kaiser Permanente Northern California (KPNC), an integrated health care system serving over 4.5 million members. We studied adults (aged ≥18 years) with T2D in the KPNC diabetes registry, identified using diagnoses, medications, and laboratory results based on an internally validated (100% sensitivity, 97% specificity) algorithm.^18,19^ From a cohort of active KPNC members with T2D, patients were classified as high risk for hypoglycemia using our validated 6-factor risk-stratification tool^20,21,22^ or as intermediate risk with a hypoglycemia-related ED or IP encounter in the prior year and sulfonylurea use. The hypoglycemia risk-stratification tool uses 6 clinical factors (number of prior episodes of hypoglycemia-related utilization, insulin use, sulfonylurea use, prior year ED use, chronic kidney disease stage, and age) to categorize patients as low, intermediate, or high risk for hypoglycemia-related ED visits or hospitalizations. Exclusion criteria included dementia, psychosis, end-stage kidney disease, skilled nursing facility residence, no primary care clinician, no contact preference, concurrent enrollment in another deprescribing study, or death before outreach, leaving a final eligible sample of 1204 patients. Among those, a cohort was randomly selected on June 1, 2023, and assigned 1:1 to intervention or usual care. Power calculations that justified this sample size were previously published.^17^ We then conducted a structured medical record review to exclude the small number of remaining ineligible patients. In the intervention arm, we removed 4 patients (3 with T1D and 1 with end-stage kidney disease). In the usual care arm, we removed 5 patients (4 with T1D and 1 who was no longer a KPNC member) (Figure). The trial protocol and statistical analysis plan can be accessed in Supplement 1. The Kaiser Foundation Research Institute institutional review board approved the study and classified it as quality improvement, waiving the requirement for informed consent. This study followed the Consolidated Standards of Reporting Trials (CONSORT) reporting guideline.

**Figure.**
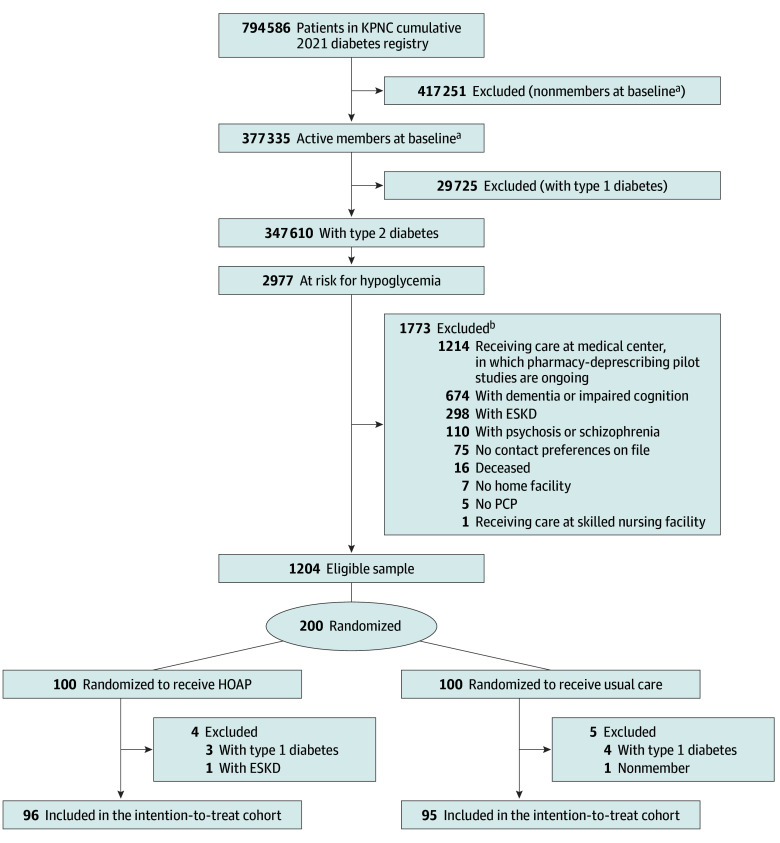
CONSORT Diagram of Patients ESKD indicates end-stage kidney disease; HOAP, Hypoglycemia on a Page; KPNC, Kaiser Permanente Northern California; PCP, primary care physician. ^a^Baseline was July 23, 2023. ^b^Exclusion numbers do not sum to 1773 because some patients were excluded for multiple criteria.

The intervention was delivered by a pharmacist (M.W.C.) with clinical diabetes expertise who reviewed intervention-arm patient medical records, obtained primary care team approval to contact the patient, and then conducted proactive outreach. Initial telephone encounters (45 minutes) assessed hypoglycemia history, risk factors, medication use, and glycemic data. Guided by the HOAP algorithm, the pharmacist set individualized glycemic targets; optimized medications (discontinuing sulfonylureas, mealtime [bolus] insulin, or mixed insulin when appropriate); provided education on hypoglycemia prevention and treatment; and made health education, nutrition, or social services referrals as needed. Trained medical phone interpreters were used for all patients with limited English proficiency. Follow-up calls (15-30 minutes) occurred every 1 to 4 weeks until hypoglycemia resolved, based on patient-reported symptoms, in addition to review of blood glucose levels, or the trial ended (January 22, 2024). Patients in the usual care arm did not receive proactive outreach by the study pharmacist but rather standard diabetes care at the discretion of the primary care team.

The primary outcome was prescribing a safer diabetes regimen at 6 months, defined by discontinuation of sulfonylureas and/or mealtime (bolus) or mixed insulin. Given the relatively rare occurrence of severe hypoglycemia (approximately 6.7% per year in this population at high risk^20^), we did not have sufficient power to examine this as the primary outcome with the limited timeline for this study. However, evidence from prior studies has demonstrated that diabetes medication deprescribing is directly associated with a reduction of hypoglycemic events over time.^23^ We did not include discontinuation of long-acting insulin in the definition for the primary outcome because we presumed that the majority of patients would still require basal insulin therapy.^24^ This aligns with published guidelines for managing patients with severe hypoglycemia.^25^ We also did not include medication dose adjustments because these are often not accurately documented.

Secondary outcomes included glucagon prescribing (emergency treatment for hypoglycemia), continuous glucose monitor (CGM) prescribing, hypoglycemia documentation in the electronic medical record problem list, HbA_1c_ less than 8% (to convert percentage of total HbA_1c_ level to proportion of total hemoglobin, multiply by 0.01), and hypoglycemia-related ED or IP utilization at 6 months. Data were extracted directly from a secure, internal KPNC reporting database that captures prescribing orders, clinical notes, laboratory results, and diagnoses; all outcome measures were collected from data available during routine care. Measures of glucagon and CGM prescribing and documentation of hypoglycemia in the problem list were collected on the last day of the intervention (January 22, 2024). For each patient, we collected the last HbA_1c_ recorded in the 6 months after the intervention. Likewise, we counted the number of hypoglycemia events in the 6 months after the intervention (defined by a primary diagnosis from an ED encounter or a principal diagnosis from an IP admission using *International Statistical Classification of Diseases, Tenth Revision, Clinical Modification* diagnosis codes).^21^ The only outcome that had missing data was HbA_1c_. We also evaluated the HbA_1c_ level and hypoglycemia-related ED or IP utilization at 12 months as an exploratory outcome (post hoc) to assess durability of the intervention.

### Statistical Analysis

Analyses followed the intention-to-treat (ITT) principle. We compared baseline characteristics across treatment arms using standardized differences, which express the magnitude of the difference between groups in units of the pooled SDs. For primary and secondary outcomes, we present risk differences (RDs) between study arms and calculated 95% CIs for the RD using the Miettinen-Nurimen method.^26^ Prespecified subgroup analyses assessed heterogeneity by age (<75 years vs ≥75 years), self-reported race and ethnicity, and eligibility criteria. Race and ethnicity categories included Asian, Black, Latino, White, and other (American Indian or Alaska Native, Native Hawaiian or Other Pacific Islander, and multiracial or multiethnic). We hypothesized that baseline risk or treatment response between race and ethnicity groups may differ and therefore included that assessment in our analytic plan. We stratified by age 75 years because the care protocols within this health system differ based on that age cutoff, with population-level oversight of younger patients (aged <75 years) and primary care physician–led oversight of older patients (aged ≥75 years). In addition, we tested for interactions between eligibility criteria or race and ethnicity and study arm. Statistical analyses were performed using SAS, version 9.4M7 (SAS Institute Inc) with a significance level of 2-sided *P* < .05.

## Results

Among 347 610 active KPNC members with T2D, 2977 were classified as high risk for hypoglycemia, and after exclusions, the final eligible sample included 1204 patients. From those patients, 200 were enrolled and randomly selected; assigned to the 2 arms; and after further exclusions, the ITT cohort included 191 patients (96 intervention, 95 usual care). The mean (SD) age was 71.3 (11.5) years (100 females [52.4%] and 91 males [47.6%]). In the intervention arm, 73 patients completed an initial visit, 32 completed a second visit, and 19 completed 3 or more visits. Of the eligible patients in the intervention arm, there was a mean (SD) 3.23 (1.24) encounters per patient. The intervention arm included patients who were slightly older than the usual care arm (mean [SD] age, 73.8 [10.4] years vs 68.7 [12.1] years) (Table 1). Of the total cohort, 20 patients (10.5%) were Asian, 34 (17.8%) were Black, 33 (17.3%) were Latino, 90 (47.1%) were White, and 12 (6.3%) were of other race or ethnicity; 10 (5.2%) reported limited English proficiency.

**Table 1.** Baseline Characteristics of the Eligible Study Population in the Intention-to-Treat Analysis^a^

Variable	Patients, No. (%)		
	Total (N = 191)	Intervention arm (n = 96)	Usual care arm (n = 95)
Age, mean (SD), y	71.3 (11.5)	73.8 (10.4)	68.7 (12.1)
Age, y			
<75	113 (59.2)	50 (52.1)	63 (66.3)
≥75	78 (40.8)	46 (47.9)	32 (33.7)
Race and ethnicity			
Asian	20 (10.5)	10 (10.4)	10 (10.5)
Black	34 (17.8)	18 (18.8)	16 (16.8)
Latino	33 (17.3)	20 (20.8)	13 (13.7)
White	90 (47.1)	41 (42.7)	49 (51.6)
Other^b^	12 (6.3)	6 (6.3)	6 (6.3)
Missing	2 (1.1)	1 (1.0)	1 (1.1)
HbA_1c_ level, mean (SD), %	8.2 (1.6)	8.0 (1.3)	8.3 (1.8)
HbA_1c_ <8%	95 (51.6)	46 (51.1)	49 (52.1)
Chronic kidney disease stage 4 or 5	22 (11.5)	13 (13.5)	9 (9.5)
Sex^c^			
Female	100 (52.4)	52 (54.2)	48 (50.5)
Male	91 (47.6)	44 (45.8)	47 (49.5)
Limited English proficiency	10 (5.2)	5 (5.2)	5 (5.3)
Hypoglycemia-related ED or IP encounter in the past 12 mo	94 (49.2)	44 (45.8)	50 (52.6)
≥3 Episodes of hypoglycemia-related utilization in the past 3 y	6 (3.1)	1 (1.0)	5 (5.3)
Prescribed CGM	90 (47.1)	41 (42.7)	49 (51.6)
Prescribed glucagon	7 (3.7)	3 (3.1)	4 (4.2)
Hypoglycemia noted on the problem list	31 (16.2)	12 (12.5)	19 (20.0)
Eligibility pathways			
High hypoglycemia risk score	173 (90.6)	87 (90.6)	86 (90.5)
ED visit or hospitalization for hypoglycemia and sulfonylurea dispensed in the past 12 mo	18 (9.4)	9 (9.4)	9 (9.5)
Prescriptions for diabetes medications at baseline^d^			
Sulfonylurea	46 (24.1)	25 (26.0)	21 (22.1)
Insulin	167 (87.4)	82 (85.4)	85 (89.5)
Basal insulin	157 (82.2)	75 (78.1)	82 (86.3)
Bolus insulin	107 (56)	50 (52.1)	57 (60)
Mixed insulin	12 (6.3)	5 (5.2)	7 (7.4)
Sulfonylurea and/or bolus insulin and/or mixed insulin	141 (73.8)	68 (70.8)	73 (76.8)
Metformin	78 (40.8)	38 (39.6)	40 (42.1)
SGLT2 inhibitor	27 (14.1)	14 (14.6)	13 (13.7)
GLP-1 agonist	24 (12.6)	10 (10.4)	14 (14.7)
Thiazolidinedione	3 (1.6)	0	3 (3.2)

Abbreviations: CGM, continuous glucose monitoring; ED, emergency department; GLP-1, glucagon-like peptide-1; HbA_1c_, hemoglobin A_1c_ (glycated); IP, inpatient; SGLT2, sodium-glucose cotransporter-2.

SI conversion factor: To convert percentage of total HbA_1c_ level to proportion of total hemoglobin, multiply by 0.01.

^a^ Baseline characteristics were measured at randomization (June 1, 2023), with the exception of prescribed CGM, prescribed glucagon, and hypoglycemia noted on the problem list, which were measured on July 19, 2023, 1 day before the intervention began on July 20, 2023. Data are reported as No. (%) of patients unless otherwise indicated.

^b^ Includes American Indian or Alaska Native, Native Hawaiian or Other Pacific Islander, and multiracial or multiethnic.

^c^ Study patients only included females and males.

^d^ Basal insulin (intermediate or long acting) and bolus insulin (short or rapid acting) were counted as separate therapeutic classes; the other 8 classes were alpha-glucosidase inhibitors, metformin, dipeptidyl peptidase IV inhibitors, GLP-1 agonists, meglitinides, SGLT2 inhibitors, sulfonylureas, and thiazolidinediones. There were no orders for the following diabetes medications at baseline: alpha-glucosidase inhibitors, dipeptidyl peptidase IV inhibitors, or meglitinides.

The mean (SD) baseline HbA_1c_ level was 8.2% (1.6%) (intervention: 8.0 [1.3]; control: 8.3 [1.8]), and the number (percentage) of insulin users was 167 (87.4%) (intervention: 82 [85.4%]; control: 85 [89.5%]) and of sulfonylurea users was 46 (24.1%) (intervention: 25 [26.0%]; control: 21 [22.1%]). Approximately half of the patients (94 [49.2%]) had a hypoglycemia-related ED or IP encounter in the prior 12 months, yet only 31 (16.2%) had hypoglycemia documented on the problem list at baseline. Glucagon prescriptions were rare (7 [3.7%]), although CGM prescriptions were common (90 [47.1%]).

Most patients (173 [90.6%]) met eligibility criteria for study inclusion due to a high-risk hypoglycemia score. Hypoglycemia-prone medications were commonly prescribed at baseline: 46 (24.1%) were prescribed a sulfonylurea; 157 (82.2%), basal insulin; 107 (56.0%), bolus (mealtime) insulin; and 12 (6.3%), mixed insulin. Metformin (78 [40.8%]) was also frequently prescribed. Less common were sodium-glucose cotransporter-2 inhibitors (27 [14.1%]), glucagon-like peptide-1 receptor agonists (24 [12.6%]), and thiazolidinediones (3 [1.6%]). No patients were prescribed alpha-glucosidase inhibitors, dipeptidyl peptidase IV inhibitors, or meglitinides.

At the end of the trial, prescription of a safer diabetes regimen (primary outcome) was more frequent in the intervention group compared with usual care (27 [28.1%] vs 15 [15.8%]; RD, 12.3% [95% CI, 0.6%-24.0%]) (Table 2). Of note, the contributors to safer diabetes regimens, in order of frequency, included the discontinuation of bolus insulin (31 [73.8%]), followed by the discontinuation of a sulfonylurea (12 [28.6%]), and then the discontinuation of mixed insulin (3 [7.1%]). Some patients discontinued more than 1 therapy.

**Table 2.** Primary and Secondary Outcomes at Follow-Up in the Intention-to-Treat Analysis^a^

Outcome	Patients, No. (%)		Risk difference (95% CI), %^b^
	Intervention arm (n = 96)	Usual care arm (n = 95)	
**Primary**			
Prescribed safer diabetes regimen	27 (28.1)	15 (15.8)	12.3 (0.6 to 24.0)
**Secondary**			
Prescribed CGM	54 (56.3)	52 (54.7)	1.5 (−12.5 to 15.5)
Prescribed glucagon	16 (16.7)	5 (5.3)	11.4 (2.7 to 20.8)
Hypoglycemia noted on the problem list	63 (65.6)	21 (22.1)	43.5 (30.1 to 55.3)
HbA_1c_ <8% (6 mo)	47 (61.8)	49 (63.6)	−1.8 (−17.0 to 13.5)
HbA_1c_ level, mean (SD), %	8.0 (1.6)	7.9 (1.7)	0.1 (−0.4 to 0.6)
Hypoglycemia-related ED or IP utilization (6 mo)	0	5 (5.3)	−5.3 (−11.8 to −1.3)

Abbreviations: CGM, continuous glucose monitoring; ED, emergency department; HbA_1c_, hemoglobin A_1c_ (glycated); IP, inpatient.

SI conversion factor: To convert percentage of total HbA_1c_ level to proportion of total hemoglobin, multiply by 0.01.

^a^ Measured on January 22, 2024 (trial end date), except HbA_1c_ and hypoglycemia-related ED or IP utilization. HbA_1c_ was the last measure during the 6 months after baseline (January 23 to July 22, 2024). Hypoglycemia-related ED or IP utilization was collected during the 6 months after baseline (January 23 to July 22, 2024). Data are reported as No. (%) of patients unless otherwise indicated.

^b^ 95% CIs were calculated using the Miettinen-Nurimen method.^26^

At randomization, 50 of 191 patients (26%) already met the primary outcome condition of being prescribed a safer regimen (ie, not treated with sulfonylureas, bolus, or mixed insulin), with no differences between the intervention and usual care arms (28 [29.2%] vs 22 [23.2%]). This may have been due to either deprescribing by the primary care team prior to randomization or the patient already being treated with only basal insulin. These patients were eligible for inclusion in the study based on their high hypoglycemia risk score and thus were included in the ITT analysis. When restricting to the subset of patients treated with a sulfonylurea, bolus insulin, or mixed insulin at randomization, the difference in the primary outcome was even larger (27 [40%] in the intervention arm vs 15 [21%] in the usual care arm; RD, 19.2% [95% CI, 4.0% to 33.7%]).

We found significant differences by trial arm for 3 of the secondary outcomes (Table 2). Glucagon prescribing was more frequent in the intervention arm (16 [16.7%] vs 5 [5.3%]; RD, 11.4% [95% CI, 2.7% to 20.8%]), as was documentation of hypoglycemia on the problem list (63 [65.6%] vs 21 [22.1%]; RD, 43.5% [95% CI, 30.1% to 55.3%]). None of the patients in the intervention arm experienced hypoglycemia-related ED or IP utilization at 6 months compared with 5 (5.3%) of the usual care patients (RD, −5.3% [95% CI, −11.8% to −1.3%]) (Table 2). By 12 months, the frequency of hypoglycemia-related ED or IP utilization remained lower in the intervention group compared with the usual care group, although the difference between groups was no longer significant; 1 patient (2.1%) in the intervention arm vs 6 patients (6.3%) in usual care had experienced hypoglycemia-related ED or IP utilization (RD, −4.2% [95% CI, −11.3% to 1.8%]). There were no differences in CGM prescribing between intervention and usual care arms (54 [56.3%] vs 52 [54.7%]; RD, 1.5% [95% CI, −12.5% to 15.5%]) (Table 2). Furthermore, glycemic control (HbA_1c_ <8%) did not differ between groups at 6 months (47 [61.8%] vs 49 [63.6%] in intervention and usual care arms; RD, −1.8% [95% CI, −17.0% to 13.5%]) (Table 2) or at 12 months (56 [58.3%] vs 56 [58.8%]; RD, −0.5% [95% CI, −15.2% to 14.3%]). Similarly, the mean (SD) HbA_1c_ level did not differ between treatment groups at 6 months (8.0% [1.6%] vs 7.9% [1.7%] in intervention and usual care arms; RD, 0.1% [95% CI, −0.4% to 0.6%]) (Table 2) or at 12 months (8.0% [1.7%] vs 8.1% [2.0%]; RD, −0.2% [95% CI, −0.7% to 0.4%]). There were 38 patients (20 in the intervention and 18 in the usual care arm) with missing HbA_1c_ data at 6 months.

In age-stratified analyses (Table 3), there was no difference in rates of safer prescribing by study arm for patients younger than 75 years (9 [18.0%] vs 11 [17.5%]; RD, 0.5% [95% CI, −13.6% to 15.6%]); however, prescription of a safer diabetes regimen was more frequent in the intervention group compared with usual care for those aged 75 years or older (18 [39.1%] vs 4 [12.5%]; RD, 26.6% [95% CI, 6.7% to 43.8%]). CGM prescriptions did not differ significantly by arm in either age group. Glucagon prescriptions were twice as high in the intervention group for those younger than 75 years (11 [22.0%]) compared with the intervention group for those aged 75 years or older (5 [10.9%]); however, the RD between study arms within each age strata was of a similar order of magnitude: 14.1% (95% CI, 1.1% to 28.5%) among those younger than 75 years and 10.9% (95% CI, −0.5% to 23.1%) among those aged 75 years or older, albeit, the former was statistically significant and the latter was not. Documentation of hypoglycemia on the problem list was more frequent in the intervention group compared with usual care in both age strata (<75 years: 30 [60.0%] vs 17 [27.0%]; RD, 33.0% [95% CI, 14.8% to 49.3%]; ≥75 years: 33 [71.7%] vs 4 [12.5%]; RD, 59.2% [95% CI, 38.9% to 73.8%]). Neither HbA_1c_ less than 8% at 6 months nor hypoglycemia-related ED or IP encounters at 6 months differed between groups in either age stratum. We tested for interactions between eligibility criteria or race and ethnicity and study arm and did not find evidence of effect modification.

**Table 3.** Primary and Secondary Outcomes at Follow-Up, Stratified by Age, in the Intention-to-Treat Analysis^a^

Outcome by age	Patients, No. (%)		Risk difference (95% CI), %^b^
	Intervention arm (n = 96)	Usual care arm (n = 95)	
**Primary**			
Prescribed safer diabetes regimen			
<75 y	9 (18.0)	11 (17.5)	0.5 (−13.6 to 15.6)
≥75 y	18 (39.1)	4 (12.5)	26.6 (6.7 to 43.8)
**Secondary**			
Prescribed CGM			
<75 y	31 (62.0)	41 (65.1)	−3.1 (−20.9 to 14.6)
≥75 y	23 (50.0)	11 (34.4)	15.6 (−6.9 to 36.2)
Prescribed glucagon			
<75 y	11 (22.0)	5 (7.9)	14.1 (1.1 to 28.5)
≥75 y	5 (10.9)	0	10.9 (−0.5 to 23.1)
Hypoglycemia noted on the problem list			
<75 y	30 (60.0)	17 (27.0)	33.0 (14.8 to 49.3)
≥75 y	33 (71.7)	4 (12.5)	59.2 (38.9 to 73.8)
HbA_1c_ <8% (6 mo)			
<75 y	29 (64.4)	36 (66.7)	−2.2 (−21.0 to 16.3)
≥75 y	18 (58.1)	13 (56.5)	1.5 (−24.3 to 27.7)
Hypoglycemia-related ED or IP utilization (6 mo)			
<75 y	0	3 (4.8)	−4.8 (−13.1 to 2.6)
≥75 y	0	2 (6.3)	−6.3 (−20.3 to 1.8)

Abbreviations: CGM, continuous glucose monitoring; ED, emergency department; HbA_1c_, hemoglobin A_1c_ (glycated); IP, inpatient.

^a^ Measured on January 22, 2024 (trial end date), except HbA_1c_ and hypoglycemia-related ED or IP utilization. HbA_1c_ was the last measure during the 6 months after baseline (January 23 to July 22, 2024). Hypoglycemia-related ED or IP utilization was collected during the 6 months after baseline (January 23 to July 22, 2024).

^b^ 95% CIs were calculated using the Miettinen-Nurimen method.^26^

## Discussion

In this randomized clinical trial, patients in the proactive, pharmacist-led intervention arm were approximately twice as likely as those in the usual care arm to be prescribed a safer diabetes regimen at 6 months (28.1% vs 15.8%). In age-stratified analyses, this finding was driven by more deprescribing in the older age stratum (≥75 years [39.1% vs 12.5%]). This study took place in a population, which, at baseline, already demonstrated a high risk for severe hypoglycemia; approximately 50% of patients in this study had experienced a hypoglycemia-related ED or IP encounter in the past 12 months. This rate of hypoglycemia-related utilization is approximately 100-fold higher than that seen in the general T2D population (approximately 0.5%).^21,27^ In addition, 91% of study patients were designated as high risk, according to our validated hypoglycemia risk-stratification tool,^20,21,22^ indicating a greater than 5% predicted 12-month risk of any hypoglycemia-related utilization. Despite this high predicted risk, 74% of patients in both groups were still using at least 1 higher-risk medication for hypoglycemia (sulfonylureas, bolus insulin, or mixed insulin) at the time of randomization, and only 3% to 4% of patients were prescribed glucagon, indicating a substantial opportunity for further intervention. Notably, even though 26% of patients in the ITT analysis had medications deprescribed prior to randomization, significant between-group differences still emerged in prescribing safer diabetes regimens and in hypoglycemia-related health care utilization.

Not all patients could be deprescribed, despite high hypoglycemia risk. Barriers reported by patients and the study pharmacist included the need for continued intensive glycemic control (eg, due to symptomatic hyperglycemia), patient preference, and clinical judgment regarding safety of medication changes. These findings highlight the challenges of implementing a uniform deprescribing algorithm. Even with a structured, evidence-based protocol such as HOAP, medication adjustments required a shared decision-making process and individualization based on comorbidities, treatment goals, patient preferences, and readiness for change.

Importantly, safer prescribing in the intervention arm resulted in no hypoglycemia-related ED or IP encounters in the 6 months after the intervention and no deterioration in HbA_1c_ control, suggesting improved safety without loss of efficacy. In a separate unpublished study, we analyzed a natural experiment to assess whether the May 2023 dissemination of the new hypoglycemia guideline HOAP across KPNC was associated with a reduction in rates of ED visits and hospitalizations for hypoglycemia. We were unable to detect a significant effect, suggesting that dissemination of a guideline, without proactive identification and outreach to patients at risk, may not change prescribing patterns of diabetes physicians. These results support expanding team-based care models to incorporate protocol-driven, proactive pharmacist outreach for patients at high risk.

### Strengths and Limitations

An important strength of this study is that it was performed in a health system with broad representation of race and ethnicity, educational level, and socioeconomic status. Because the study population is similar to the general US population, our study findings are likely generalizable outside of this health care system. Additionally, we had access to extensive population-level data, with very high ascertainment, in the setting of an integrated health care model that combines health coverage and care delivery, allowing us to clearly define patient characteristics and outcomes.

This study also has limitations. Results must be interpreted within the limitations of the study design. The intervention was given by a single pharmacist and performed within a health system, in which midlevel clinicians conduct most diabetes management based on physician-approved treatment protocols. It is not clear whether a similar intervention would have the same impact in a system that does not use team-based care to the same extent. At baseline, the intervention arm was slightly older than the usual care arm. This may have explained some of the differential prescribing patterns in the primary outcome. Additionally, because severe hypoglycemic events are rare and underreported in medical records, we had limited power to detect between-group differences in the secondary outcome. A previous study has shown that hypoglycemia-related ED or IP encounters documented in the medical records represent a small fraction (approximately 5%) of severe hypoglycemia events,^10^ most of which occur at home and are underreported to health care practitioners. Future studies to investigate this outcome may need to be larger or have more intensive data-collection protocols by research staff.

## Conclusions

In this randomized clinical trial, proactive outreach by a clinical pharmacist applying the HOAP deprescribing protocol led to significantly safer diabetes medication regimens among adults with T2D at high risk for hypoglycemia, particularly among those prescribed hypoglycemia-prone medication regimens (sulfonylureas, bolus insulin, or mixed insulin). This intervention improved medication safety without worsening glycemic control and resulted in fewer hypoglycemia-related acute care encounters. Embedding structured, pharmacist-led deprescribing strategies into team-based care is an effective, scalable approach to reducing treatment-related harm in populations at high risk.

## Notes

Supplement 1.
Trial Protocol and Statistical Analysis Plan

Supplement 2.
Data Sharing Statement
