# Supplementary material for: Pharmacist Intervention for Safer Prescribing in Patients With Type 2 Diabetes at High Risk: A Randomized Clinical Trial
Source: JAMA Netw Open. 2026 Feb 18;9(2):e2559946. doi: 10.1001/jamanetworkopen.2025.59946 (PMC12917679; doi:10.1001/jamanetworkopen.2025.59946)
Supplement: Supplement 1. — Trial Protocol and Statistical Analysis Plan [file jamanetwopen-e2559946-s001.pdf]

**INSTRUCTIONS:**

- Please ensure you are using the most recent version available in IRBNet.
- Complete this Protocol Template only when there is **no** existing authored protocol provided for this study.
- If you are conducting a data-only study with no prospective or interventional components, use the Data Only Protocol Template instead.
- This Protocol Template is to be used in conjunction with the SMART KP IRB Core Data Form.
- Enter your responses to each question directly below the BLUE text in the fillable field.
- When completing this Protocol Template, if a section does not apply to your study then enter "N/A."

1. **Protocol**

   Protocol Title

   **Testing a new population management model for hypoglycemia prevention in high-risk KPNC members.**

   Principal Investigator

   Lisa Gilliam, MD, PhD
   Richard Grant, MD, MPH

   Version Date

   October 27, 2022

   Form Author

   Howard Moffet

2. **Objectives**

   Describe in plain language the purpose, specific aims, or objectives and indicate the primary goal(s) of the study (e.g. safety, tolerability, effectiveness, feasibility, pilot study, etc.). State the hypotheses to be tested. State primary and any secondary study endpoints.

   The primary research question is whether using a "Hypoglycemia Champion" (HC) to target high risk patients with type 2 diabetes (T2D) is a feasible and effective model of care to reduce hypoglycemia risk in this population. The HC will be a specially trained accountable population manager (APM) who will implement a new clinical guideline intended to reduce risk of hypoglycemia.

   The aims of this study are to:

   1. Convene meetings with stakeholders (endocrinologists within and outside of KP) to develop an evidence-based, expert consensus clinical guideline, "Hypoglycemia on a Page" (HOAP), for hypoglycemia prevention in patients with T2D at high risk of hypoglycemia; publish HOAP in the KPNC clinical library and disseminate for general use by KPNC diabetes care providers (APMs and PCPs) via regional presentations as the new standard of care;
   2. Develop a workflow/clinical pathway for use by a "Hypoglycemia Champion" (HC), a clinical pharmacist/accountable population manager (APM) ("HC APM") who will proactively outreach and

apply the HOAP guideline in the management of KPNC members identified as having high hypoglycemic risk;

3. Conduct an evaluation of whether proactive application of the HOAP clinical pathway by the HC APM is associated with better diabetes regimen safety among KPNC members at high risk of hypoglycemia compared to patients receiving usual APM care (including use of HOAP). We hypothesize that at 6 months, high-risk members assigned to the HC APM will be prescribed safer diabetes regimens compared to usual care.

**Note:** We are currently seeking IRB approval to conduct the first 2 aims of the study (**Phase 1**), which do not involve any patient contact or any research-related activities. This **Phase 1** of the study involves development work with content experts. Given the compressed timeline for this award, we need IRB approval now to begin Phase 1.

We will submit a modification for Aim 3 (**Phase 2**) after completion of **Phase 1**, when we will have a final version of our implementation plan. We will not conduct any Aim 3 activities before IRB approval of this planned IRB modification.

## 3. Background

a. Scientific Background

Provide the scientific or scholarly background for, rationale for, and significance of the research based on the existing literature and how will it add to existing knowledge. A list of references or bibliography must be included as part of this document or uploaded separately.

Hypoglycemia in patients with type 2 diabetes (T2D) is a significant adverse drug event associated with the hypoglycemia-inducing diabetes medications, insulin and sulfonylureas. Hypoglycemia has been poorly addressed by health plans and all but ignored by national performance metrics such as HEDIS.

Hypoglycemia is common (in a survey of 20,188 diabetes patients from KPNC, 12% reported having ≥1 severe hypoglycemia events requiring third-party assistance in the previous 12 months), morbid (associated with falls, fall-related fractures, automobile accidents, cardiovascular events, dementia, and even death), and costly (total annual direct medical costs ~$1.8 billion in 2009 in the US). Hypoglycemia often leads to significant diabetes distress and nonadherence to prescribed diabetes medications. Providers are challenged to address the competing demands of prescribing glucose-lowering medications to meet glycemic targets while avoiding hypoglycemia.

In addition, there are now strategic considerations for reducing hypoglycemia risk. NCQA is proposing a new HEDIS measure assessing emergency department visits for hypoglycemia in older adults with diabetes for measurement year 2023 (MY2023). There is little time to prepare before its proposed implementation in 2023. Unlike most of the existing HEDIS measures, this new measure is focused on patients >65 years of age and is not capped at 75 years of age, which matches the age range where hypoglycemia risk is highest. Hypoglycemia risk can be mitigated, but there exist no well-validated, population management approaches for hypoglycemia prevention. Our proposal intends to address this gap by developing and evaluating a practical population management approach to hypoglycemia prevention.

Severe hypoglycemia is the most common adverse drug effect associated with diabetes medications. However, hypoglycemia in patients with T2D is not adequately recognized, documented, or treated by

care providers. Ever since landmark clinical trials (e.g., UKPDS) demonstrated that lowering blood glucose levels reduced the risk of long-term diabetes complications, the medical community has prioritized A1C reduction, often underestimating the risks of diabetes medications, especially hypoglycemia. Each year, approximately 11% of KPNC patients with type 2 diabetes experience severe hypoglycemia (a hypoglycemic episode requiring third-party assistance), with the risk increasing with age and duration of diabetes.(Karter, 2018) In older adults, approximately 13% of emergency department admissions for adverse drug events are due to hypoglycemia (primarily in insulin-treated patients)(Shehab, 2016), and annual hospital admissions for hypoglycemia now surpass those for hyperglycemia.(Lipska, 2014)

The research question is whether an intervention by a hypoglycemia champion directed towards patients at high risk of hypoglycemia can reduce hypoglycemia events more than usual care (which will include a new treatment algorithm aimed at hypoglycemia risk reduction).

We previously developed a hypoglycemia risk tool which can stratify patients into low, intermediate or high risk for ED visit or hospitalization for hypoglycemia.(Karter, 2017; Karter, 2019) We demonstrated that patients classified as high risk for hypoglycemia were 35 times more likely to have an ED or hospital visit for hypoglycemia in the subsequent 12 months, compared to patients classified as low risk. However, it is important to note that ED or hospital visits for hypoglycemia are just the tip of the iceberg: 95% of patients experiencing severe hypoglycemia are cared for outside of the medical system (i.e., assisted by family or others).(Karter, 2018)

Currently, no well-validated, population management approach for hypoglycemia prevention exists. Front line clinicians need information and guidance on how to minimize hypoglycemia risk while optimizing glycemic control to reduce long-term diabetes complications. Thus, the development and evaluation of interventions to address hypoglycemia risk are needed and are highly relevant to TPMG care delivery, patient safety, and strategic goals of maintaining stellar HEDIS performance metrics.

This work has strategic implications for our organization because a new HEDIS measure proposed for measurement year 2023 will assess emergency department admissions for hypoglycemia in older adults with diabetes. Our proposal will address this gap in organizational clinical care by developing and evaluating a practical, proactive, population management approach to hypoglycemia prevention for members with T2D at high risk of hypoglycemia

b. Preliminary Data

Describe any relevant preliminary data.

None

4. **Study Design**

Describe the overall approach of the study (e.g. prospective, interventional, observational, retrospective, etc.). If your study includes more than one group, arm, or subject population, describe that here (for example, a study of both subjects and their caregivers, or a study with both a prospective interventional arm and a retrospective chart review arm).

**Phase 1 (not Human Subjects Research)**

In **Phase 1** of this study (Aims 1 & 2), we will:

- convene meetings with endocrinologists to develop and publish a new hypoglycemia prevention guideline, "Hypoglycemia on a Page" (HOAP) [Aim 1], and
- develop a clinical workflow for a clinical pharmacist/APM Hypoglycemia Champion ("HC APM") to proactively apply the new guideline [Aim 2]

Aim 1 – Develop and disseminate "Hypoglycemia on a Page" (HOAP)

- Develop an evidence-based hypoglycemia prevention algorithm to improve the safety of medication regimens for patients with T2D. As with prior guidelines (e.g., "PHASE on a Page"), we will convene regional and national stakeholders to review evidence, gather clinical insights, integrate feedback, and reach consensus for a single standard clinical care guideline for hypoglycemia prevention in high-risk members across KPNC.
- Disseminate HOAP regionally for use by KPNC diabetes care providers (APMs and PCPs)

Aim 2 – Develop the workflow protocol for use by the "Hypoglycemia Champion" (HC) APM, a clinical pharmacist who will be trained to apply the HOAP guideline to the proactive management of members at high risk of hypoglycemia. This structural change will be modeled after our highly effective PHASE cardiovascular risk management program.

**We are currently requesting IRB approval to conduct Phase 1 of this 2-year proposal. Further protocol details, screenshots of the final tool, and other materials required for Phase 2 will be submitted to the IRB at a later date as a modification. No work on the second phase of the project will be done until IRB approval has been obtained in the modification.**

**Phase 2 (modification will be submitted at a later date)**

In **Phase 2** of this study (Aim 3), we will:

- conduct a prospective evaluation of whether assignment to the Hypoglycemia Champion is associated with better diabetes regimen safety among KPNC members at high risk of hypoglycemia compared to usual care [Aim 3].

Aim 3 –Conduct a prospective evaluation of whether assignment to the Hypoglycemia Champion APM is associated with better diabetes medication regimen safety among KPNC members at high risk of hypoglycemia compared to usual care.

- Randomly assign eligible study participants to the HC APM for comparison to data-only matched controls (receiving usual care subsequent to the regionwide dissemination of HOAP as new standard of care). For all participants or data-only matched controls, all medical decisions are individually made and approved by clinicians responsible for member care. Primary Outcome: Proportion of high-risk patients prescribed safer diabetes regimens (discontinuation at 6 months compared to pre-baseline of sulfonylureas, mealtime [rapid-/short-acting], or mixed insulin) comparing study arms. We hypothesize that more patients in the intervention arm will be prescribed safer diabetes regimens. Baseline pharmacy dispensing data and medication discontinuation orders during follow up will be collected directly from EHR/Clarity tables.
    i. As researchers, we will not be contacting patients directly at any time.

ii. All care will be delivered under approved protocols by KPNC clinicians.
iii. All evaluation data related to clinical outcomes will be collected directly from the medical record of usual care.

- Secondary Outcomes: We will also examine differences between participants and data-only matched patients in glucagon prescribing, CGM use, hypoglycemia on the patient's problem list, HbA1c<8% (HEDIS-defined glycemic control metric), and ED visits or hospitalizations for hypoglycemia based on primary diagnosis. We hypothesize that participants will have more glucagon prescribing, CGM use, and inclusion of hypoglycemia on the problem list, with stable glycemic control (HbA1c<8%), and fewer ED visits or hospitalizations for hypoglycemia. We will also conduct Heterogeneity of Treatment Effect (HTE) analyses by age (<75 vs. ≥ 75 years), enrollment criterion (hypoglycemia prediction algorithm vs ED/hospital hypoglycemia admission), and race/ethnicity.

Assess whether this study involves a clinical trial. Clinical trial means a research study in which one or more human subjects are prospectively assigned to one or more interventions (which may include placebo or other control) to evaluate the effects of the interventions on biomedical or behavioral health-related outcomes.

**Phase 2 (modification will be submitted at a later date)**
While not a clinical trial (since all patients will be offered usual care), Aim 3 will include a prospective evaluation of whether random assignment to the Hypoglycemia Champion (usual care delivered by an APM trained in the new standard of care) is associated with better diabetes regimen safety among KPNC members at high risk of hypoglycemia compared to usual care (usual APM care management). All KPNC patients at high risk for hypoglycemia are expected to receive the new HOAP protocol delivered by their usual APM. Outcomes among participants in Aim 3 will compared to a data-only matched sample receiving usual care.

Describe whether the study involves educational tests, survey procedures, or interview procedures.

N/A

Describe whether the research involves benign behavioral interventions on **adult** subjects. Note: Benign behavioral interventions are brief in duration, harmless, painless, not physically invasive, not likely to have a significant adverse lasting impact on the subjects, and not offensive or embarrassing to the subjects. Examples include having subjects play online games or solving puzzles under various noise conditions.

N/A

If the study involves either educational tests, survey procedures, interview procedures, or benign behavioral interventions **(on adults)**, specify whether one of the following criteria is met:

(i) The information obtained is recorded by the investigator in such a manner that the identity of the human subjects cannot readily be ascertained, directly or through identifiers linked to the subjects

N/A

(ii) Any disclosure of the human subjects' responses outside the research would not reasonably place the subjects at risk of criminal or civil liability or be damaging to the subjects' financial standing, employability, educational advancement, or reputation

N/A

(iii) The information obtained is recorded by the investigator in such a manner that the identity of the human subjects can readily be ascertained, directly or through identifiers linked to the subjects, and an IRB conducts a limited IRB review to make the determination

N/A

**5. Study Population**

a. Number of Subjects

State the number of subjects you plan to include at the KP region to which this study is being submitted. If applicable, distinguish between the number of subjects who are expected to be enrolled/screened and the number of subjects needed to complete the research procedures (e.g. number of subjects excluding screen failures).

**Phase 1**
Aim 1 meeting participants are not research subjects. They are collaborators with subject matter expertise. Aim 2 is an activity with no research subjects.

**Phase 2 (modification will be submitted at a later date)**
Aim 3 will enroll up to 100 subjects ("participants") from KPNC and compare them to an equal number of matched data-only patients.

As appropriate, differentiate between different populations of subjects within the same study (e.g. subject/caregiver, parent/child, patient/physician).

In this study, the only subjects are patients ("participants") in Aim 3 receiving care from the HC APM.

If this is a multicenter study, indicate the total number of subjects to be accrued across all sites.

N/A

If this study has a data only component, indicate the total number of patient records to be accessed.
Control subjects for Aim 3 will have 100 matched subjects for data-only.

b. Inclusion and Exclusion Criteria

- Describe the criteria that define who will be included or excluded in your final study sample.

**Phase 2 (modification will be submitted at a later date)**

Aim 3 study participants will include patients with type 2 diabetes (T2D) at high risk for hypoglycemia, including those 1) designated as "high-risk" by the existing hypoglycemia risk stratification tool[1, 2] developed by our team and currently implemented into Health Connect, or 2)

who have had an ED visit or hospitalization for hypoglycemia (as primary or principal diagnosis, respectively) in 6 months prior to cohort baseline (date of enrollment in cohort).

No exclusion criteria.

- Describe how individuals will be screened for eligibility.

**Phase 2 (modification will be submitted at a later date)**
The data analyst will identify a cohort of eligible patients from the KPNC Diabetes Registry based on the inclusion and exclusion criteria. 100 study participants will be randomly selected; 100 matched data-only patients will be selected.

- If you are planning to conduct remote informed consent, how will you assure that participants should not be excluded from the study? e.g. how will you determine that potential participants can understand, adequately hear, have appropriate decision making capacity, and have a copy of the informed consent form?

**Phase 2 (modification will be submitted at a later date)**
We request a waiver of informed consent.

- If information or biospecimens will be obtained for the purpose of screening, recruiting, or determining eligibility, informed consent is not required if one of the following criteria is met. Select one of the following criteria and provide a brief explain for how the criteria is met.

  ☐ The investigator will obtain information through oral or written communication with the prospective subject or legally authorized representative, or

  ☐ The investigator will obtain identifiable private information or identifiable biospecimens by accessing records or stored identifiable biospecimens

  N/A

  IMPORTANT NOTES: Although informed consent may not be required, HIPAA Privacy Authorization may still be required. Also, if this study is FDA-regulated, then consent may be required.

- Describe the plan for disposition of information/biospecimens collected during recruitment/screening in the event of a screen failure or when a potential subject is contacted but declines participation (e.g., destroyed immediately, destroyed at end of study, retained for separate analysis or so that subjects are not contacted repeatedly about participation after they have declined, etc.).

N/A

c. Subjects Vulnerable to Coercion or Undue Influence

Indicate whether you will include or exclude each of the following special populations. Justify the inclusion of any of these populations. Describe additional safeguards to protect the rights and welfare of

these subjects. Note: This refers to subjects who are known members of these populations upon enrollment or at any time during the study.

- Children

  Study will not include children.

- Neonates of uncertain viability or nonviable neonates (up to 28 days post birth)

  N/A

- Prisoners (NOTE: The KP IRB does not have the appropriate membership to review research involving prisoners. Consultation with the IRB Office will be required.)

IMPORTANT NOTE: Consider whether subjects will be in a vulnerable category at the time of information/biospecimen collection or during analysis. For instance, if you collect information/biospecimens about children who were ages 12 – 15 from years 2000 – 2002, you know that now those individuals are no longer children.

Individuals with Impaired Decision-Making Capacity

Indicate how you will assess decision making capacity and cognitive function. This process must be tailored based on the risk and design of the study.

**Phase 2 (modification will be submitted at a later date)**
We will not assess decision-making capacity or cognitive function.

State whether individuals with impaired decision-making capacity will be included.

N/A

Explain the extent of cognitive impairment (complete, fluctuating, progressive, or temporary).

N/A

Justify their inclusion and explain any protections to mitigate risk (such as the involvement of a caregiver or legally authorized representative).

N/A

Describe the process to determine whether an individual is capable of consent, and submit any documents that will be used assess decisional capacity.

N/A

List the individuals from whom permission will be obtained in order of priority. (E.g., durable power of attorney for health care, court appointed guardian for health care decisions, spouse, and adult child.

N/A

Describe the process for assent of the subjects by addressing the following:

- Whether assent will be required of all, some, or none of the subjects. If assent will be obtained from some subjects, indicate which subjects will be required to assent and which will not.

  N/A

- If assent will not be obtained from some or all subjects, an explanation of why not.

  N/A

- When assent is obtained, describe how it will be documented.

  N/A

HIV Status: N/A

If the study will be ascertaining subject HIV status for study exclusion/inclusion, please indicate how:

☐ Prospective laboratory HIV testing of subjects for the study

☐ Surveying subjects about their HIV status

☐ KPNC HIV Registry access or existing electronic health record data*

*If you checked this option, you must first gain approval from the HIV Steering Committee by contacting Michael J. Silverberg, PhD, MPH. The HIV Steering Committee approval must be submitted to IRBNet.

Will TPMG physicians be directly contacted to enroll as study participants? ☐ **Yes** ☒ **No**

**If "yes" the contact must be approved by Yi Fen Irene Chen, MD, Associate Executive Director, TPMG, prior to IRB review. Dr. Chen's approval must be submitted to IRBNet.**

Other Populations Targeted for Recruitment
If you are targeting a population that may be vulnerable to coercion or undue influence based on the specific circumstances of the study, describe how you will ensure that participation is voluntary and minimize any added risk. (Common examples include employees, students, economically or educationally disadvantaged persons, etc.)

N/A

d. Setting
Describe the sites or locations where your research team will conduct the research.

**Phase 1**

Aim 1: These small group meetings will be held virtually using a KP IT approved application.

Aim 2: The workflow will be developed under the direction of the PIs at SSF and DOR.

**Phase 2 (modification will be submitted at a later date)**
Aim 3: The HC APM will be based in SSF; data analysis will be conducted at the Division of Research.

If this is a multi-site study:

- Specify what procedures are being performed at this site or by this site's personnel (consider recruitment, consent process, study procedures, information/biospecimen analysis, etc.).

  N/A

- State how each site will satisfy its IRB review requirements. Indicate if you are asking this site's IRB to rely on another IRB or if another institution would like to rely on this site's IRB and include this information in the eIRB Initial Project submission

  N/A

For research conducted outside this site describe: (Community, Reservations etc.)

- Describe site-specific regulations or customs affecting the research at that location.

  N/A

- Local scientific and ethical review structure outside this site.

  N/A

**6.** Recruitment Methods
Describe how study participants will be recruited and enrolled.

**Phase 2 (modification will be submitted at a later date)**
There will be no recruitment. All participants will already be receiving care from an APM and will be assigned the HC as their new APM.

Indicate whether you will openly recruit using advertisements, websites, flyer, or brochures. (Upload the final versions of all recruitment materials to your submission to the IRB.)

N/A

Indicate if you plan to do targeted recruitment using existing records or referral. Please submit final versions of all referral emails/scripts.
N/A

Describe, by position/title, who will be recruiting and enrolling participants (providing the specific names of research team members is not necessary).

**Phase 2 (modification will be submitted at a later date)**

Participants will be enrolled by the Hypoglycemia Champion who will be a pharmacist APM.

Describe any plans for the participants in the currently proposed study to be re-contacted or recruited for future follow-up studies. (Note that participants should be informed of this potential for re-recruitment or future follow-up studies during the current study's consent process.)

None.

Please note the following KPNC IRB guidelines:

- Contact of prospective subjects will be limited to three (3) attempts in one week, for no longer than three (3) continuous weeks.
- Messages left will be limited to one (1) per week for no more than three (3) weeks.
- No more than two (2) recruitment mailings (email, flyer, brochure, etc.).
- If permission from the patient's PCP is necessary to contact the patient:
  - If PCP does not respond to request, recruitment attempts (phone, mailings, etc.) may not begin until 2 weeks after the PCP's permission was requested.
  - If PCP grants permission, recruitment attempts may begin with no waiting period.

**7.** Informed Consent Process

a. Written Consent

Describe how you will obtain and document consent, including:

**Phase 2 (modification will be submitted at a later date)**

We seek a waiver of written informed consent.

- Where, when and how the consent process will take place.

  N/A

- How the research team will ensure that subjects have sufficient time to consider whether to participate in the research

  N/A

- A process to ensure ongoing consent.

  N/A

- Steps that will be taken to minimize the possibility of coercion or undue influence.

  N/A

- Any steps that will be taken to ensure the subjects' understanding.

  N/A

- If you are planning to conduct remote informed consent, please describe the process in detail, including any electronic platforms. Indicate how the consent process will be documented.

  N/A

NOTE: For each federally-supported clinical trial, one IRB-approved informed consent form used to enroll subjects must be posted by the awardee or sponsor conducting the trial on a publicly available Federal website that will be established as a repository for such informed consent forms.

b. Waiver of Informed Consent

Provide rationale and justification for the Waiver of Informed Consent for this study, including:

- Explain how the proposed research presents no more than minimal risk to the study participants.

**Phase 2 (modification will be submitted at a later date)**

Participants will receive usual care from the HC APM (whose goal is to fully implement the new standard of care protocol with the intention of reducing the risk of hypoglycemia and improving safety). The attention presents no more than minimal risk to participants.

- Explain how the waiver of informed consent will not adversely affect the rights and welfare of the participants.

**Phase 2 (modification will be submitted at a later date)**

Receiving usual care with proactive attention from the HC APM will not affect the rights and welfare of participants.

- Explain why this research cannot practically be carried out without a waiver of informed consent. Note: research regulations require that justification for a waiver of consent explain why it is impracticable to perform the research, and not just impracticable to obtain consent. Practicability cannot be determined solely by considerations of convenience/cost/speed.

**Phase 2 (modification will be submitted at a later date)**

Receipt of usual care does not require obtaining consent at every step of every encounter. To introduce a consent requirement into the APM encounter would be inappropriate.

- If the research involves using identifiable private information or identifiable biospecimens, provide justification for why the research cannot practicably be carried out using deidentified information.

**Phase 2 (modification will be submitted at a later date)** The research could not be practicably carried out using de-identified information or data because linking the data extracted from the sources requires a common identifier to link the data.

- Assess whether it is appropriate to provide the subjects with additional pertinent information after participation.

**Phase 2 (modification will be submitted at a later date)**

In the course of usual care, patients are provided with pertinent information about their care and care options.

c. Waiver of Signed (Documented) Informed Consent

Provide rationale and justification for the Waiver of Signed (Documented) Informed Consent by identifying which of these three conditions applies and justification for how the criteria is met.

1) The research involves no more than minimal risk to participants AND involves no procedures for which written consent is normally required outside of the research context.

**Phase 2 (modification will be submitted at a later date)**

Participants will receive usual care from an APM (whose goal is to fully implement the new standard of care protocol) which does not normally require written consent.

2) The signed consent form would be the only record linking the participants to the research, and the principal risk to participants would be potential harm resulting from a breach of confidentiality.

N/A

3) The subjects or legally authorized representatives are members of a distinct cultural group or community in which signing forms is not the norm.
    a. Describe the distinct cultural group or community
    b. Describe why signing forms is not the norm
    c. Explain how the research presents no more than minimal risk of harm to these subjects
    d. Provide an appropriate alternative mechanism for documenting that informed consent is obtained

N/A

d. Alteration of Informed Consent

Identify the required elements of informed consent that you wish to remove or alter.

N/A

Provide justification for their removal or alteration.

N/A

e. Non-English-Speaking Subjects

If subjects who do not speak English will be enrolled, describe how the consent discussion will take place and indicate if translated consent forms or short forms will be used. Confirm that an interpreter will assist with the initial consent process and subsequent study visits.

All contact with non-English-Speaking patients will utilize interpreters, in accordance with the usual clinical workflows when care is provided to these patients. As previously noted, we will seek a waver of signed consent, since this is not different from usual clinical care.

IMPORTANT NOTE: Please be aware that if it is expected that you will enroll non-English speakers in the study, short forms should not be used as the only Consent option for these individuals, they should only be used if a non-English speaking participant is unexpectedly encountered. However, the possibility of encountering non-English speaking potential subjects in the Bay Area is a possibility and this possibility should be considered, and budgeted for, when preparing the initial study submission for IRB review.

f. Assent of Children and Parent Permission

IMPORTANT NOTE: Child Consent may be obtained in certain situations (for example, conducting family planning or sexually transmitted disease (STD) research). In addition, for older children ages 16 and up who participate in an adult study, the consent document can be used in place of the assent document.

Describe how you will obtain and document assent/parental permission, including:

- Describe your plan for obtaining parent permission. The permission of one parent is generally sufficient for minimal risk research, or for greater than minimal risk research if there is the potential for direct benefit to the child. For studies involving greater than minimal risk with no prospect of direct benefit to the child, permission of both parents is required unless one parent is deceased, unknown, incompetent, or not reasonably available, or when only one parent has legal responsibility for the care and custody of the child.

  N/A Children will not be enrolled.

- Describe whether permission will be obtained from individuals other than parents, and if so, who will be allowed to provide permission.

  N/A

- Indicate whether assent will be obtained and documented from all, some, or none of the children.

  N/A

- If assent will only be obtained from some children (because of very young age, severe cognitive impairment, etc.), indicate which children will be required to assent and which will not.

  N/A

- When assent of children is obtained, describe whether and how it will be documented.

  N/A

- When subjects might reach the age of majority during the study, describe the plan to obtain consent from these subjects at that time using an adult consent form.

  N/A

g. Secondary Research for Which Consent is Not Required:

Note: Research involving the use of identifiable biospecimens does not apply to this section.
Note: Although consent may not be required, justification for a waiver of HIPAA Privacy Authorization may still be required.

Secondary Research of identifiable private information or identifiable biospecimens may not require informed consent if at least one of the criteria listed below is met. Select at least one of the following criteria and provide a rationale for how the criteria is met:

- Use of publicly available identifiable private information or identifiable biospecimens.

  N/A

- The information and/or biospecimens are recorded by the investigator in such a way that the identity of the subjects cannot be readily ascertained, and the investigator will neither contact the subjects nor re-identify subjects.

  N/A

- The research involves only information collection (i.e., no biospecimen collection) and the analysis of this identifiable health information is regulated by HIPAA.

  Data will be collected on data-only matched controls (receiving usual care subsequent to the regionwide dissemination of HOAP).

**8. HIPAA Privacy Rule Authorization**

a. Written HIPAA Privacy Rule Authorization:
Describe the plan to obtain a signed Privacy Rule Authorization from each subject.

**Phase 2 (modification will be submitted at a later date)**
We request waiver of HIPAA authorization.

b. Waiver of HIPAA Privacy Rule Authorization
If you will not obtain a signed HIPAA Privacy Rule Authorization or if you want to eliminate any required language from the authorization, provide the following rationale and justification.

- Explain why the research could not practicably be conducted without the waiver. Note: research regulations require that justification for a waiver of HIPAA Authorization explain why it is impracticable to perform the *research*, and not just impracticable to obtain HIPAA Authorization. Practicability cannot be determined solely by considerations of convenience/cost/speed.

  Receipt of usual care does not require obtaining HIPAA Privacy Rule Authorization at every step of every encounter. To introduce a consent requirement into the APM encounter would be inappropriate.

- Explain why access to and use of the PHI is necessary for the research.

It is necessary to access PHI to assemble the cohort. Without access to and use of the necessary PHI the research cannot be conducted. Direct patient contact is necessary for usual care as well as for this activity.

- Explain why the use or disclosure of PHI for the research poses no more than minimal risk to the subjects' privacy.

Use of PHI poses no more than minimal risk as this study will store PHI in password protected or encrypted electronic files and stored on protected KP servers within each region and within KP networks secured by firewalls. Passwords will not be disclosed to any party not involved in the study, including study teams outside of the KP NCAL region. Patient identifiers will not appear in any part of the publications. When it is necessary to share PHI information among study parties within each respective KP region, we will transmit the files (password-protected/encrypted) through encrypted KP email or secure file transfer. For all these reasons the use and disclosure of PHI poses no more than minimal risk to the subjects' privacy.

- Provide an adequate plan to protect the PHI from improper use or disclosure.

This study will store PHI in password protected or encrypted electronic files and stored on protected KP servers within KP network secured by firewalls. Passwords will not be disclosed to any party not involved in the study outside of each region. Patient identifiers will not appear in any part of the publications. When it is necessary to share PHI information among study parties within the immediate KP regional team, we will transmit the files (password-protected/encrypted) through encrypted KP email or secure file transfer. Only designated trained study staff within each regional study team will have access to all data collected as part of this study.

- Provide a plan to destroy identifiers at the earliest opportunity consistent with the purpose of the research.

Personal identifiers associated with participants will be destroyed as soon as possible after the study has finished and after publication of all manuscripts. Since manuscript submission and revision process often requires re-access of patient information, patient identifiers will be kept until all publications generated from the study have been finished, at which time all patient identifiers will be destroyed at the close of the study.

c. **HIPAA Disclosure Accounting**

The Health Insurance Portability and Accountability Act (HIPAA) Privacy Rule gives patients the right to receive a listing, known as an accounting of disclosure, of their information that is disclosed to others for reasons other than treatment, payment, or health care operations. KP must account for all known disclosures of protected health information for research purposes without the individual's authorization (a waiver or alteration of HIPAA Authorization) both within a KP region (between a Permanente Medical Group, Kaiser Foundation Hospitals, and a Kaiser Foundation Health Plan) and outside of KP.

- What type of PHI is being disclosed?
  ☒ Clinical/Diagnostic
  ☒ Demographics
  ☒ Healthcare

- How many participants are expected to be enrolled in this study at your region?
  ☐ 49 or less individuals

  The investigator should maintain an individual accounting record of all Disclosures made by the research team that are subject to the HIPAA tracking requirements. The investigator must also transmit this tracking information within 20 days of Disclosure to the Regional Compliance Officer. Using: Health Connect Quick Disclosure or Complete the Disclosure of PHI about a single individual for a research purpose Form.

  ☒ 50 or more individuals

  If 50 or more individuals, please provide the information for each entity that sponsored the research where PHI is being disclosed. *Please note: If you are disclosing PHI to more than one entity, the following is needed for each entity.*

  - **Name**
    KPNC Region
  - **Address**
    2000 Broadway, Oakland, CA 94612
  - **Phone Number**
    510-891-3400

If disclosing PHI to more than one entity, please continue entering the required information below:
PHI will not be disclosed outside of the KP NCAL entity.

**9. Study Procedures**

a. Description

Describe and explain the study design, including:

- A detailed chronological description of all research procedures.

**Phase 1:**

Aim 1

Months 1-4: Meetings with stakeholders will be convened by PIs.
Months 4-6: Finalize and disseminate "Hypoglycemia on a Page" (HOAP) (such as dissemination at all regional meetings with APMs, "steal this talk" presentations for PCPs, and electronic posting in the Clinical Library).

Aim 2

Months 2-4: Workflow/clinical pathway will be developed by PIs.

**Phase 2 (modification will be submitted at a later date)**

Aim 3

Months 3-6:

- Develop procedures and outcome metrics (e.g. hypoglycemia questionnaires) for outcome evaluation
- Identify the study cohort (N=200)(have a high hypoglycemia risk score or a recent ED visit or hospitalization for hypoglycemia); randomly select participants and data-only matched sample
- Collect baseline EMR data on study cohort

Months 7-12:

- HC APM conducts outreach to study participants for assessment and evaluation; collects baseline self-reported outcome data (e.g., fear of hypoglycemia) and provides hypoglycemia intervention.

**NOTE: After the development of the workflow (Aim 2), a study Modification will be submitted to IRB to provide the details of the HC APM process.**

Months 13-21

- Hypoglycemia champion collects follow-up data outcome data (e.g., re-administer the outcome metrics 6 months after baseline) from participants

Months 21-24

- Analyze data and draft manuscript

- Procedures to monitor subjects for safety, including who will review the data and at what frequency for safety issues.

**Phase 2 (modification will be submitted at a later date)**

APM will routinely monitor participants for safety and report any concerns to the Investigator.

- Procedures performed to lessen the probability or magnitude of risks.

**Phase 2 (modification will be submitted at a later date)** Usual care has no more than minimal risk.

- The source records that will be used to collect information about subjects. (Attach all surveys, scripts, and data collection forms.)

**Phase 2 (modification will be submitted at a later date)**

Data will be collected from EMR.

Additional data will be collected by the APM HC from self-reported outcomes (e.g., fear of hypoglycemia) and provides hypoglycemia intervention, etc.

- What information and/or biospecimens will be collected including during long-term follow-up.

*Please note, if biospecimens being collected are being used to develop an investigational in vitro diagnostic device, an IDE for the device may be needed. Please review the IVD Device FAQs in IRBNet for guidance.*

**Phase 2 (modification will be submitted at a later date)**

Data will be collected from EMR (glucagon prescribing and dispensing, CGM use (device and supply prescribing and dispensing, hypoglycemia on the patient's problem list, HbA1c results, and ED visits or hospitalizations for hypoglycemia based on primary diagnosis.

Additional data will be collected by the APM HC from self-reported outcomes (e.g., fear of hypoglycemia) and provides hypoglycemia intervention, etc.

- The duration of an individual subject's participation in the study.

**Phase 2 (modification will be submitted at a later date)**

Participation in the study will end within 12 months after enrollment of first participant and no later than 12/31/2024.

- The duration anticipated to enroll all study subjects.

  Enrollment is expected to end by 01/31/2024.

- The estimated date for the investigators to complete this study (complete primary analyses) NOTE: It should be clear exactly which procedures will be conducted for the research as opposed to procedures the subjects would undergo (in the exact manner described in the protocol) even if they were not participating in the study.

  The study is expected to be completed by 12/31/2024.

- Describe procedures that will be followed when subjects withdraw from the research, including withdrawal from intervention but continued information and/or biospecimen collection.
  Participants who become non-adherent to care provided by HC APM will be followed up per the intent to treat analysis plan.

- Describe any anticipated circumstances under which subjects could be withdrawn from the research without their consent.

  Discontinuation of Kaiser membership.

- Describe any procedures for orderly termination.

  N/A

- If the study involves genetic testing or collection of genetic information, describe this.

N/A

- Clarify whether research involving biospecimens will (if known) or might include whole genome sequencing (i.e., sequencing of a human germline or somatic specimen with the intent to generate the genome or exome sequence of that specimen).

  N/A

- Does the study include basic and clinical research involving recombinant or synthetic nucleic acid molecules, including the creation and use of organisms and viruses containing recombinant or synthetic nucleic acid molecules, and is subject to NIH guidelines (Guidelines: https://osp.od.nih.gov/biotechnology/nih-guidelines/ and FAQs: https://osp.od.nih.gov/biotechnology/faqs-on-ibc-administration/)?

  ☐ Yes
  If yes, your study may require Institutional Biosafety Committee Review. Please contact kpnc.irb@kp.org for guidance.

  ☒ No

  Optional comments:
  N/A

b. Data Analysis
Describe the data analysis plan, including:

- Statistical procedures.

**Phase 2 (modification will be submitted at a later date)**
Statistical Design: The primary analysis for Aim 3 will be an intent-to-treat analysis using chi square tests and binomial regression. For the primary outcome, we will test for differences in the proportion of patients prescribed safer diabetes regimens (i.e., discontinuation of sulfonylureas, mealtime or mixed insulin) at 6 months in the treatment vs. control arms. For the secondary outcomes, we will test for differences in the proportion of patients who had an ED visit or hospitalization for hypoglycemia within 6 months, were prescribed CGM or prescribed glucagon, and were in glycemic control (HbA1c<8%) between the 2 study arms. We will perform exploratory Heterogeneity of Treatment Effect (HTE) analyses for age, race and enrollment criterion (see above) by testing for statistical interactions with the exposure (treatment arm).

- When applicable, the power analysis.

**Phase 2 (modification will be submitted at a later date)**
Power Calculation: In preparation for this proposal, we evaluated baseline use of hypoglycemia-prone medications (e.g., sulfonylureas, mealtime, or mixed insulin) in 3921 KPNC patients identified as "high risk" by our hypoglycemia risk tool. Among these patients, 75% were on hypoglycemia-prone treatment. We estimate that 60% of the intervention arm and 20% of the

usual care arm will be prescribed safer diabetes regimens within 6 months compared to baseline. The minimum number of subjects needed to detect this 40% absolute difference, assuming 90% power and a 10% attrition rate, is 86 patients (43 per arm). With an enrollment size of 200 (100 per arm), this trial is more than adequately powered to detect the hypothesized difference.

- Any procedures that will be used for quality control of collected data.

  ?

c. Sharing of Results with Subjects

Describe whether results (study results or individual subject results, such as results of standard or research lab tests and genetic tests) will be shared with subjects or their providers and under which circumstances.

N/A

If the study carries a risk of incidental findings, describe your plan for evaluating these and determining whether and how subjects or their providers will be given this information.
N/A

If laboratory results will be shared with subjects or their healthcare providers, verify that the laboratory conducting the test is Clinical Laboratory Improvement Amendments (CLIA) certified.
N/A

Describe how study participants will be updated on research progress via newsletter, summary, etc. Consider incorporating such updates to study participants as part of ongoing retention efforts.
N./A

**10. Privacy, Confidentiality and Data Security**

Describe the steps that will be taken to protect subjects' privacy during recruitment, consent and study procedures.

Usual care procedures for protecting patient privacy will be used.

Describe the plan for storage of data and/or biospecimens including:

- Who will have access?

The PI, Co-I's and study team will have access to identifiable information from the medical record and other clinical databases. PHI will not be disclosed outside of study team.

- Where the data/materials will be stored and for how long. Indicate if data will be encrypted and password protected and if transportable/removable media will be used. Indicate if data will be stored on non-KP devices or sponsor-provided devices.

Data will be stored in password protected or encrypted electronic files and stored on protected KPNC servers within KP network secured by firewalls. Passwords will not be disclosed to any party

not involved in the study. Patient identifiers will not appear in any part of the publications. When it is necessary to share PHI information among study parties within KPNC, we will transmit the files (password-protected/encrypted) through encrypted KPNC email or secure file transfer. The information will be stored for the length of the project and destroyed after the final manuscripts are published. Data will be NOT stored on non-KP devices or sponsor-provided devices.

- If applicable, how will data be transmitted? (e.g. Encrypted email, secure file transfer, sponsor provided site)

Data will be transmitted via password-protected/encrypted files through encrypted KPNC email or secure file transfer.

- What identifiers will be included.

  MRNs, dates

- Any other steps that will be taken to ensure security (e.g., training of staff, authorization of access, password protection, encryption, physical security, and separation of identifiers from data and specimens, certificates of confidentiality).

  None, we believe our plan to protect the data is appropriately mitigated.

- Describe the plan to destroy/archive or retain data at the end of the study. If storing information and/or identifiable biospecimens for future research, complete the next section.

  Personal identifiers associated with participants will be destroyed as soon as possible after the study has finished and after publication of all manuscripts. Since manuscript submission and revision process often requires re-access of patient information, patient identifiers will be kept until all publications generated from the study have been finished, at which time all patient identifiers will be destroyed at the close of the study.

## 11. Information and/or Biospecimen Banking for Future Research

If you are creating a repository, please submit a separate protocol.

Indicate if biospecimens may be used for future research and whether that may include genetic research.

N/A

State if information or biospecimens will be sent to a separate repository. If data or specimens will be banked in a repository for future use as part of this protocol submission address the following questions:

- What will be banked and what identifiers will be associated with the information or biospecimens?

N/A

- Where and how will the information or specimens be stored?

  N/A

- For what purpose will the information or specimens be used? Include a general description of the types of research that may be conducted with the identifiable private information/biospecimens (e.g. research on cancer).

  N/A

- How will the information or specimens be accessed, and who will have access?

  N/A

- Describe the procedures to release information or specimens, including the process to request a release, approvals required for release, who can obtain information or specimens, and the information to be provided with specimens.

  N/A

- How long will identifiable private information/biospecimens be stored?

  N/A

**12. Collection of data from subjects electronically**
If you will collect any data from participants electronically (including email, website, etc.), explain:

- Does the study involve a Mobile Device or Application? If yes, please indicate the source/manufacturer/developer of the product:

  No.

- How and what data will be collected. Indicate if PHI is collected electronically (including via an app) and if there is any interface with the subject's personal accounts (for example: if subjects need to create an account to use the device, or if the device interfaces with an external source of data, or medical record).

  N/A

- How the information will be secured/transmitted/stored (encryption, password protection, etc.; may require consultation with IT department). Include a data flow diagram if data will flow through multiple parties (such as a hosting provider or coordinating center).

  N/A

- Any risks to the participants' privacy posed by using these methods (describe in consent, as applicable).

  N/A

- Describe if email containing PHI will be used to communicate with participants? (Per KP policy, securing messaging must be used for all communications containing PHI.)

  N/A

- How you will verify the participant's identity.

  N/A

**13. Disclosure of PHI to a collaborator**

If any data will be sent outside of this site, list each recipient (may list by role or category if the information is the same for several different entities). For each recipient, describe:

- The name and location of the individual/entity receiving the information.

  N/A

- What information and/or biospecimens will be sent.
  N/A

- Whether the information will be fully identifiable (PHI, if health information), a Limited Data Set, de-identified, or aggregate.

  N/A

- How the data/materials will be transferred securely (for instance, Secure File Transfer). Indicate if hard copy PHI will be stored/sent to a collaborator.

  N/A

- When applicable, clarify whether there are written assurances from collaborators that PHI will not be reused or re-disclosed to any other entity. Describe assurances that PHI will be stored securely. Describe mechanisms (e.g., intrusion detection software or regular electronic system activity audits or monitoring) for determining if KP PHI has been inappropriately or illegally accessed, used, disclosed, or modified.

  N/A

**14. Provisions to Monitor Data to Ensure the Safety of Subjects**

This is required when research involves more than Minimal Risk to subjects.

The plan might include establishing a data monitoring committee and a plan for reporting data monitoring committee findings to the IRB and the sponsor. Describe:

- Who will monitor the study data for safety?

  N/A. This study involves no more than minimal risk to subjects.

- The plan to periodically evaluate the data collected regarding both harms and benefits to determine whether subjects remain safe.

  N/A

- What data are reviewed, including safety data, untoward events, and efficacy data.

  N/A

- How the safety information will be collected (e.g., with case report forms, at study visits, by telephone calls with participants).

  N/A

- The frequency of data collection, including when safety data collection starts.

  N/A

- The frequency or periodicity of review of cumulative data.

  N/A

- Criteria for taking action on monitoring findings (for instance, stopping rules, immediate suspension, reporting, protocol changes, changes to monitoring frequency or plan).

  N/A

- For studies monitored by a DSMB/C, describe the committee membership and structure, meeting format, and quorum requirements.  Upload the board/committee charter, if one exists.

  N/A

**15. Risks and Benefits**

a. Risks to Subjects

List the reasonably foreseeable risks, discomforts, hazards, or inconveniences to the subjects related the subjects' participation in the research. Consider physical, psychological, social, legal, and economic risks.

We do not anticipate any foreseeable risks, discomforts, hazards, or inconveniences to participants.

Describe the probability, magnitude, duration, and reversibility of the risks.
N/A

If applicable, indicate which procedures may have risks to the subjects that are currently unforeseeable.

N/A

If applicable, indicate which procedures may have risks to an embryo or fetus should the subject be or become pregnant.

N/A

If applicable, describe risks to others who are not subjects and risks to Kaiser Permanente

N/A

b. Potential Benefits to Subjects
Describe the potential benefits that individual subjects may experience from taking part in the research. Include as may be useful for the IRB's consideration, the probability, magnitude, and duration of the potential benefits. Indicate if there is no direct benefit. Do not include benefits to society or others.

The study is intended to reduce risk of hypoglycemia and improve patient safety. Participants may experience these benefits.

c. Risks to KP
Is there anything about the nature of this study which, if revealed to the public, could put KP at risk or competitive disadvantage? ☐ **Yes** ☒ **No**
If "yes" describe in detail.

**16. Economic Burden to Subjects**
Describe any costs that subjects may be responsible for because of participation in the research study (for example, co-pays; paying for treatment, therapies, or other interventions, or the delivery of these) and how you will inform participants of these costs prior to their enrollment in this study.

None

**17. Compensation to Participants**
Describe any compensation provided to participants, for example, for time inconvenience, discomfort, travel, or in the event of research related injury. If applicable, describe how you will inform participants of this prior to their enrollment in the study, including if payment will be prorated if the subject withdraws early from the study.

NOTE: payment may not be withheld as an incentive for participants to complete any portion of the study.

None.

**18. Resources Available**
Describe any special resources or expertise required to conduct the study.

Click or tap here to enter text.

19. **Principal Investigator**
    a. Has the Principal Investigator (PI) previously been approved as PI for a study within KPNC? ☒ Yes ☐ No
    b. Has the PI been audited/assessed within the past three years? ☐ Yes ☒ No
    If Yes, check all that apply and provide the outcome/findings:
    ☐ FDA ☐ RQCC ☐ CTP ☐ CCRU
    Outcome/Findings: Click or tap here to enter text.
    c. Is the PI currently under a Corrective And Preventative Action (CAPA) plan?
    ☐ Yes ☒ No
    If Yes, provide a brief description: Click or tap here to enter text.

20. **Required Approvals**
    a. Describe any approvals that will be obtained prior to commencing the research. (e.g., school, external site. funding agency, or other KP departments). Be sure to list each KP site (for example, KP San Francisco Hospital, Division of Research, KP Oakland Reg – 1800 Harrison, etc.)

    Division of Research

    b. **Facility-Based Research:** KPNC IRB requires that the Principal Investigator (PI) obtain approval signatures at **each KPNC facility** where research activity will occur. These may include local research chair, Physician-in-Chief, departmental chair, Area Manager, information technology review (as appropriate), and Division of Research scientific review (student research).

    List each KP facility that research activity will occur and specify whose signature has been/will be obtained.

    Name of Facility: SSF

    Local Research Chair (LRC): Robert Li

    Chief of Service: Joanie Loh, MD

    Physician-in-chief (PIC): John Skerry, MD

    Area Managers (if applicable): Sheila Gilson

    c. **Non-Facility-Based Research** (ex: Northern California Regional and Program Office): For non-facility-based research, the PI's supervisor and department head are required in lieu of approvals from the Chief of Service, Physician-in-chief, and Area Manager. The Central Research Committee Chairperson serves as the LRC. As such, list each KP non-facility that research activity will occur and confirm whose signature has been/will be obtained.

    PI's Supervisor: Tracy Lieu, MD (Richard Grant)

Department Head: ?

Central Research Committee Chairperson: Michael Silverberg, MD

d. **Sites with no Local Research Chair:** For sites with no Local Research Chair, the Central Research Committee Chairperson serves as the Local Research Chair:

Site: N/A

Central Research Committee Chairperson: N/A

**21. Drugs or Devices**

NOTE: see the ICH-GCP guidance for a summary of investigator and sponsor responsibilities in clinical trials.

List all drugs and devices used in the research and the purpose of their use, and their regulatory approval status.

N/A

a. Drug Studies

If the research involves drugs and is investigator-initiated, indicate whether there is any possibility that the results will be reported to FDA (e.g. as part of a new drug application [NDA]).

N/A

If the drug is investigational (has an IND), confirm that you will comply with all applicable FDA requirements for investigators.

N/A

Confirm that you will follow applicable KP pharmacy policies and procedures.

N/A

Describe your plan for drug storage, handling, and accountability, including distribution, return, and destruction of the drug(s).

N/A

b. Device Studies:

If this is a device study and you think the device is Non-Significant Risk, include justification here or upload it as a separate document along with any available device information (instructions for use, etc.).

N/A

If the research involves devices and is investigator-initiated, indicate whether there is any possibility that the results will be reported to FDA (e.g. as part of a premarket approval application [PMA]).

N/A

If the device has an IDE or a claim of abbreviated IDE (Non-Significant Risk device), confirm that you will comply with all applicable FDA requirements for investigators.

N/A

Describe the device, the manufacturing process, and the device labeling, including safety instructions or warnings. If available, this may be addressed in separately uploaded device information (such as instructions for use).

N/A

Describe device storage, handling, and accountability, including how access to the device will be limited to appropriate personnel and how you will ensure the device will be used only for appropriate study subjects.

N/A

**22. Multi-Site Research**

a. If this is a multi-site study and you are the lead investigator or this site will be the coordinating center for any activity, describe the processes to ensure communication among sites, such as:

- All sites have the most current version of the protocol, consent document, and HIPAA authorization.

  *N/A*

- All required approvals have been obtained at each site (including approval by the site's IRB of record).

  *N/A*

- All modifications have been communicated to sites, and approved (including approval by the site's IRB of record) before the modification is implemented.

  *N/A*

- All engaged participating sites will safeguard data as required by local information security policies.

  *N/A*

- All local site investigators conduct the study appropriately.

*N/A*

b. Describe the method for communicating to engaged participating sites the following:

- Problems.

  N/A

- Interim results.

  N/A

- The closure of a study.

  N/A

c. Describe any special resources or expertise required to conduct the study.

*N/A*

**23. Community-Based Participatory Research**

Describe involvement of the community in the design and conduct of the research.

N/A

Describe your plan for ensuring that community research partners are appropriately trained in human subjects' protection.

N/A

NOTE: "Community-based Participatory Research" is a collaborative approach to research that equitably involves all partners in the research process and recognizes the unique strengths that each brings. Community-based Participatory Research begins with a research topic of importance to the community, has the aim of combining knowledge with action and achieving social change to improve health outcomes and eliminate health disparities.

**INSTRUCTIONS:**

- Please ensure you are using the most recent version available in IRBNet.
- Complete this Protocol Template only when there is **no** existing authored protocol provided for this study.
- If you are conducting a data-only study with no prospective or interventional components, use the Data Only Protocol Template instead.
- This Protocol Template is to be used in conjunction with the SMART KP IRB Core Data Form.
- Enter your responses to each question directly below the BLUE text in the fillable field.
- When completing this Protocol Template, if a section does not apply to your study then enter "N/A."

1. **Protocol**

   Protocol Title

   **Testing a new population management model for hypoglycemia prevention in high-risk KPNC members.**

   Principal Investigator

   Lisa Gilliam, MD, PhD
   Richard Grant, MD, MPH

   Version Date

   May 1, 2023

   Form Author

   Howard Moffet

2. **Objectives**

   Describe in plain language the purpose, specific aims, or objectives and indicate the primary goal(s) of the study (e.g. safety, tolerability, effectiveness, feasibility, pilot study, etc.). State the hypotheses to be tested. State primary and any secondary study endpoints.

   The primary research question is whether using a "Hypoglycemia Champion" (HC) to target high risk patients with type 2 diabetes (T2D) is a feasible and effective model of care to reduce hypoglycemia risk in this population. The HC will be a specially trained accountable population manager (APM) who will implement a new clinical guideline intended to reduce risk of hypoglycemia.

   The aims of this study are to:

   1. Convene meetings with stakeholders (endocrinologists within and outside of KP) to develop an evidence-based, expert consensus clinical guideline, "Hypoglycemia on a Page" (HOAP), for hypoglycemia prevention in patients with T2D at high risk of hypoglycemia; publish HOAP in the KPNC clinical library and disseminate for general use by KPNC diabetes care providers (APMs and PCPs) via regional presentations as the new standard of care;
   2. Develop a workflow/clinical pathway for use by a "Hypoglycemia Champion" (HC), a clinical pharmacist/accountable population manager (APM) ("HC APM") who will proactively outreach and

apply the HOAP guideline in the management of KPNC members identified as having high hypoglycemic risk;

3. Conduct an evaluation of whether usual care augmented by HC APM (augmented usual care) is associated with better diabetes regimen safety among KPNC members at high risk of hypoglycemia compared to patients receiving usual care. The study hypothesis is that at 6 months, eligible members assigned to the HC APM (augmented usual care) will be prescribed safer diabetes regimens compared to usual care (control). Care for all eligible members (usual care and augmented usual care) will include available use of the HOAP guidelines.

**3. Background**

a. Scientific Background

Provide the scientific or scholarly background for, rationale for, and significance of the research based on the existing literature and how will it add to existing knowledge. A list of references or bibliography must be included as part of this document or uploaded separately.

Hypoglycemia in patients with type 2 diabetes (T2D) is a significant adverse drug event associated with the hypoglycemia-inducing diabetes medications, insulin and sulfonylureas. Hypoglycemia has been poorly addressed by health plans and all but ignored by national performance metrics such as HEDIS.

Hypoglycemia is common (in a survey of 20,188 diabetes patients from KPNC, 12% reported having ≥1 severe hypoglycemia events requiring third-party assistance in the previous 12 months), morbid (associated with falls, fall-related fractures, automobile accidents, cardiovascular events, dementia, and even death), and costly (total annual direct medical costs ~$1.8 billion in 2009 in the US). Hypoglycemia often leads to significant diabetes distress and nonadherence to prescribed diabetes medications. Providers are challenged to address the competing demands of prescribing glucose-lowering medications to meet glycemic targets while avoiding hypoglycemia.

In addition, there are now strategic considerations for reducing hypoglycemia risk. NCQA is proposing a new HEDIS measure assessing emergency department visits for hypoglycemia in older adults with diabetes for measurement year 2023 (MY2023). There is little time to prepare before its proposed implementation in 2023. Unlike most of the existing HEDIS measures, this new measure is focused on patients >65 years of age and is not capped at 75 years of age, which matches the age range where hypoglycemia risk is highest. Hypoglycemia risk can be mitigated, but there exist no well-validated, population management approaches for hypoglycemia prevention. Our proposal intends to address this gap by developing and evaluating a practical population management approach to hypoglycemia prevention.

Severe hypoglycemia is the most common adverse drug effect associated with diabetes medications. However, hypoglycemia in patients with T2D is not adequately recognized, documented, or treated by care providers. Ever since landmark clinical trials (e.g., UKPDS) demonstrated that lowering blood glucose levels reduced the risk of long-term diabetes complications, the medical community has prioritized A1C reduction, often underestimating the risks of diabetes medications, especially hypoglycemia. Each year, approximately 11% of KPNC patients with type 2 diabetes experience severe hypoglycemia (a hypoglycemic episode requiring third-party assistance), with the risk increasing with age and duration of diabetes.(Karter, 2018) In older adults, approximately 13% of emergency department admissions for adverse drug events are due to hypoglycemia (primarily in insulin-treated

patients)(Shehab, 2016), and annual hospital admissions for hypoglycemia now surpass those for hyperglycemia.(Lipska, 2014)

The research question is whether an intervention by a hypoglycemia champion directed towards patients at high risk of hypoglycemia can reduce hypoglycemia events more than usual care (which will include a new treatment algorithm aimed at hypoglycemia risk reduction).

We previously developed a hypoglycemia risk tool which can stratify patients into low, intermediate or high risk for ED visit or hospitalization for hypoglycemia.(Karter, 2017; Karter, 2019) We demonstrated that patients classified as high risk for hypoglycemia were 35 times more likely to have an ED or hospital visit for hypoglycemia in the subsequent 12 months, compared to patients classified as low risk. However, it is important to note that ED or hospital visits for hypoglycemia are just the tip of the iceberg: 95% of patients experiencing severe hypoglycemia are cared for outside of the medical system (i.e., assisted by family or others).(Karter, 2018)

Currently, no well-validated, population management approach for hypoglycemia prevention exists. Front line clinicians need information and guidance on how to minimize hypoglycemia risk while optimizing glycemic control to reduce long-term diabetes complications. Thus, the development and evaluation of interventions to address hypoglycemia risk are needed and are highly relevant to TPMG care delivery, patient safety, and strategic goals of maintaining stellar HEDIS performance metrics.

This work has strategic implications for our organization because a new HEDIS measure proposed for measurement year 2023 will assess emergency department admissions for hypoglycemia in older adults with diabetes. Our proposal will address this gap in organizational clinical care by developing and evaluating a practical, proactive, population management approach to hypoglycemia prevention for members with T2D at high risk of hypoglycemia

b. Preliminary Data

Describe any relevant preliminary data.

None

## 4. Study Design

Describe the overall approach of the study (e.g. prospective, interventional, observational, retrospective, etc.). If your study includes more than one group, arm, or subject population, describe that here (for example, a study of both subjects and their caregivers, or a study with both a prospective interventional arm and a retrospective chart review arm).

**Phase 1 (not Human Subjects Research)**

In **Phase 1** of this study (Aims 1 & 2), the investigators will:

- convene meetings with endocrinologists to develop and publish a new hypoglycemia prevention guideline, “Hypoglycemia on a Page” (HOAP) [Aim 1], and
- develop a clinical workflow for a clinical pharmacist/APM Hypoglycemia Champion (“HC APM”) to proactively apply the new guideline [Aim 2]

Aim 1 – Develop and disseminate "Hypoglycemia on a Page" (HOAP)

- Develop an evidence-based hypoglycemia prevention algorithm to improve the safety of medication regimens for patients with T2D. As with prior guidelines (e.g., "PHASE on a Page"), the investigators will convene regional and national stakeholders to review evidence, gather clinical insights, integrate feedback, and reach consensus for a single standard clinical care guideline for hypoglycemia prevention in high-risk members across KPNC.
- Disseminate HOAP regionally for use by KPNC diabetes care providers (APMs and PCPs)

Aim 2 – Develop the workflow protocol for use by the "Hypoglycemia Champion" (HC) APM, a clinical pharmacist who will be trained to apply the HOAP guideline to the proactive management of members at high risk of hypoglycemia. This structural change will be modeled after KP's highly effective PHASE cardiovascular risk management program.

**Phase 2**

In **Phase 2** of this study (Aim 3), the investigators will:

- conduct a prospective evaluation of whether proactive intervention by the Hypoglycemia Champion (augmented usual care) is associated with better diabetes regimen safety among KPNC members at high risk of hypoglycemia compared to usual care (control) [Aim 3].

Aim 3 –Conduct a prospective evaluation of whether proactive intervention by the Hypoglycemia Champion APM (i.e., assignment to augmented usual care group) is associated with better diabetes medication regimen safety among KPNC members at high risk of hypoglycemia compared to usual care (control).

- Randomly assign eligible patients to receive proactive intervention by the HC APM (augmented usual care group) for comparison to data-only patients receiving usual care. All eligible patients will be managed according to the regionally disseminated HOAP as new standard of care). All medical decisions for all patients (augmented usual care and usual care) are made by each member's medical care team responsible for his or her care (not by study personnel).

- Primary Outcome: Proportion of high-risk patients prescribed safer diabetes regimens (discontinuation at 6 months compared to pre-baseline of sulfonylureas, mealtime [rapid-/short-acting], or mixed insulin) comparing study participants (augmented usual care) to data-only patients (usual care controls). The study hypothesis is that more study participants will be prescribed safer diabetes regimens compared to data-only patients. Baseline pharmacy dispensing data and medication discontinuation orders during follow up will be collected directly from EHR/Clarity tables.
    i. As researchers, patients will not be contacted directly at any time.
    ii. All care will be delivered under approved protocols by KPNC clinicians.
    iii. All evaluation data related to clinical outcomes will be collected directly from the medical record of usual care.
- Secondary Outcomes: the investigators will also examine differences between study participants and data-only patients in glucagon prescribing, CGM use, hypoglycemia on the

patient's problem list, HbA1c<8% (HEDIS-defined glycemic control metric), and ED visits or hospitalizations for hypoglycemia based on primary diagnosis. The study hypothesis is that study participants will have more glucagon prescribing, CGM use, and inclusion of hypoglycemia on the problem list, with stable glycemic control (HbA1c<8%), and fewer ED visits or hospitalizations for hypoglycemia. The investigators will also conduct Heterogeneity of Treatment Effect (HTE) analyses by age (<75 vs. ≥ 75 years), enrollment criterion (hypoglycemia prediction algorithm vs ED/hospital hypoglycemia admission), and race/ethnicity.

Assess whether this study involves a clinical trial. Clinical trial means a research study in which one or more human subjects are prospectively assigned to one or more interventions (which may include placebo or other control) to evaluate the effects of the interventions on biomedical or behavioral health-related outcomes.

**Phase 2**

While not a clinical trial (since all patients will be offered usual care), Aim 3 will include a prospective evaluation of whether receiving proactive intervention by the Hypoglycemia Champion (augmented care delivered by an APM trained in the new standard of care) is associated with better diabetes regimen safety among KPNC members at high risk of hypoglycemia compared to usual care (usual care). All KPNC patients at high risk for hypoglycemia are expected to receive the new HOAP protocol delivered by their usual APM. Outcomes among study participants in Aim 3 will compared to data-only patients receiving usual care.

Describe whether the study involves educational tests, survey procedures, or interview procedures.

N/A

Describe whether the research involves benign behavioral interventions on **adult** subjects. Note: Benign behavioral interventions are brief in duration, harmless, painless, not physically invasive, not likely to have a significant adverse lasting impact on the subjects, and not offensive or embarrassing to the subjects. Examples include having subjects play online games or solving puzzles under various noise conditions.

N/A

If the study involves either educational tests, survey procedures, interview procedures, or benign behavioral interventions **(on adults)**, specify whether one of the following criteria is met:

(i) The information obtained is recorded by the investigator in such a manner that the identity of the human subjects cannot readily be ascertained, directly or through identifiers linked to the subjects

N/A

(ii) Any disclosure of the human subjects' responses outside the research would not reasonably place the subjects at risk of criminal or civil liability or be damaging to the subjects' financial standing, employability, educational advancement, or reputation

N/A

(iii) The information obtained is recorded by the investigator in such a manner that the identity of the human subjects can readily be ascertained, directly or through identifiers linked to the subjects, and an IRB conducts a limited IRB review to make the determination

N/A

**5. Study Population**

a. Number of Subjects

State the number of subjects you plan to include at the KP region to which this study is being submitted. If applicable, distinguish between the number of subjects who are expected to be enrolled/screened and the number of subjects needed to complete the research procedures (e.g. number of subjects excluding screen failures).

**Phase 1**

Aim 1 meeting participants are not research subjects. They are collaborators with subject matter expertise. Aim 2 is an activity with no research subjects.

**Phase 2 )**

Aim 3 will identify 200 eligible patients receiving usual care from KPNC; half of these will be randomly allocated to receive augmented usual care (i.e., additional oversight be a Hypoglycemia Champion APM).

As appropriate, differentiate between different populations of subjects within the same study (e.g. subject/caregiver, parent/child, patient/physician).

In this study, the only research subjects are study participants in Aim 3 receiving augmented usual care from the HC APM.

If this is a multicenter study, indicate the total number of subjects to be accrued across all sites.

N/A

If this study has a data only component, indicate the total number of patient records to be accessed.
Control subjects for Aim 3 will have 100 matched subjects for data-only.

b. Inclusion and Exclusion Criteria

- Describe the criteria that define who will be included or excluded in your final study sample.

**Phase 2**

Eligibility criteria for Aim 3 (study participants and data-only patients) will include patients with type 2 diabetes (T2D) at high risk for hypoglycemia, including those 1) designated as "high-risk" by the existing hypoglycemia risk stratification tool[1, 2] developed by the investigator team and currently implemented into Health Connect, or 2) who have had an ED visit or hospitalization for hypoglycemia (as primary or principal diagnosis, respectively) in 6 months prior to cohort baseline (date of enrollment in cohort).

No exclusion criteria.

- Describe how individuals will be screened for eligibility.

**Phase 2**

The data analyst will identify a cohort of 200 eligible patients from the KPNC Diabetes Registry based on the inclusion and exclusion criteria. 100 study participants will be randomly allocated to receive augmented usual care; the remaining 100 data-only patients will be selected for comparison. No screening is needed.

- If you are planning to conduct remote informed consent, how will you assure that participants should not be excluded from the study? e.g. how will you determine that potential participants can understand, adequately hear, have appropriate decision making capacity, and have a copy of the informed consent form?

**Phase 2**

A waiver of informed consent is requested.

- If information or biospecimens will be obtained for the purpose of screening, recruiting, or determining eligibility, informed consent is not required if one of the following criteria is met. Select one of the following criteria and provide a brief explain for how the criteria is met.

☒ The investigator will obtain information through oral or written communication with the prospective subject or legally authorized representative, or

☐ The investigator will obtain identifiable private information or identifiable biospecimens by accessing records or stored identifiable biospecimens

N/A

IMPORTANT NOTES: Although informed consent may not be required, HIPAA Privacy Authorization may still be required. Also, if this study is FDA-regulated, then consent may be required.

- Describe the plan for disposition of information/biospecimens collected during recruitment/screening in the event of a screen failure or when a potential subject is contacted but declines participation (e.g., destroyed immediately, destroyed at end of study, retained for separate analysis or so that subjects are not contacted repeatedly about participation after they have declined, etc.).

N/A

c. Subjects Vulnerable to Coercion or Undue Influence

Indicate whether you will include or exclude each of the following special populations. Justify the inclusion of any of these populations. Describe additional safeguards to protect the rights and welfare of these subjects. Note: This refers to subjects who are known members of these populations upon enrollment or at any time during the study.

- Children

  Study will not include children.

- Neonates of uncertain viability or nonviable neonates (up to 28 days post birth)

  N/A

- Prisoners (NOTE: The KP IRB does not have the appropriate membership to review research involving prisoners. Consultation with the IRB Office will be required.)

IMPORTANT NOTE: Consider whether subjects will be in a vulnerable category at the time of information/biospecimen collection or during analysis. For instance, if you collect information/biospecimens about children who were ages 12 – 15 from years 2000 – 2002, you know that now those individuals are no longer children.

Individuals with Impaired Decision-Making Capacity

Indicate how you will assess decision making capacity and cognitive function. This process must be tailored based on the risk and design of the study.

**Phase 2**

Decision-making capacity or cognitive function of participants will not be assessed.

State whether individuals with impaired decision-making capacity will be included.

N/A

Explain the extent of cognitive impairment (complete, fluctuating, progressive, or temporary).

N/A

Justify their inclusion and explain any protections to mitigate risk (such as the involvement of a caregiver or legally authorized representative).

N/A

Describe the process to determine whether an individual is capable of consent, and submit any documents that will be used assess decisional capacity.

N/A

List the individuals from whom permission will be obtained in order of priority. (E.g., durable power of attorney for health care, court appointed guardian for health care decisions, spouse, and adult child.

N/A

Describe the process for assent of the subjects by addressing the following:

- Whether assent will be required of all, some, or none of the subjects. If assent will be obtained from some subjects, indicate which subjects will be required to assent and which will not.

  N/A

- If assent will not be obtained from some or all subjects, an explanation of why not.

  N/A

- When assent is obtained, describe how it will be documented.

  N/A

HIV Status: N/A

If the study will be ascertaining subject HIV status for study exclusion/inclusion, please indicate how:

☐ Prospective laboratory HIV testing of subjects for the study

☐ Surveying subjects about their HIV status

☐ KPNC HIV Registry access or existing electronic health record data*

*If you checked this option, you must first gain approval from the HIV Steering Committee by contacting Michael J. Silverberg, PhD, MPH. The HIV Steering Committee approval must be submitted to IRBNet.

Will TPMG physicians be directly contacted to enroll as study participants? ☐ **Yes** ☒ **No**

**If "yes" the contact must be approved by Yi Fen Irene Chen, MD, Associate Executive Director, TPMG, prior to IRB review. Dr. Chen's approval must be submitted to IRBNet.**

Other Populations Targeted for Recruitment
If you are targeting a population that may be vulnerable to coercion or undue influence based on the specific circumstances of the study, describe how you will ensure that participation is voluntary and minimize any added risk. (Common examples include employees, students, economically or educationally disadvantaged persons, etc.)

N/A

d. Setting
Describe the sites or locations where your research team will conduct the research.

**Phase 1**
Aim 1: These small group meetings will be held virtually using a KP IT approved application.

Aim 2: The workflow will be developed under the direction of the PIs at SSF and DOR.

**Phase 2**

Aim 3: The HC APM will be based in SSF; data analysis will be conducted at the Division of Research.

If this is a multi-site study:

- Specify what procedures are being performed at this site or by this site's personnel (consider recruitment, consent process, study procedures, information/biospecimen analysis, etc.).

  N/A

- State how each site will satisfy its IRB review requirements. Indicate if you are asking this site's IRB to rely on another IRB or if another institution would like to rely on this site's IRB and include this information in the eIRB Initial Project submission

  N/A

For research conducted outside this site describe: (Community, Reservations etc.)

- Describe site-specific regulations or customs affecting the research at that location.

  N/A

- Local scientific and ethical review structure outside this site.

  N/A

**6.** Recruitment Methods

Describe how study participants will be recruited and enrolled.

**Phase 2**

There will be no recruitment. All patients will already be receiving usual care from an APM.

- For study participants, the HC will communicate with each participant's provider (usual APM or physician) to coordinate care; the study participant will receive proactive intervention by the HC as an augmentation to their usual care.
- For the data-only patients, there will be no contact.

Indicate whether you will openly recruit using advertisements, websites, flyer, or brochures. (Upload the final versions of all recruitment materials to your submission to the IRB.)

N/A

Indicate if you plan to do targeted recruitment using existing records or referral. Please submit final versions of all referral emails/scripts.
N/A

Describe, by position/title, who will be recruiting and enrolling participants (providing the specific names of research team members is not necessary).

**Phase 2**
Participants will be enrolled by the Hypoglycemia Champion who will be a pharmacist APM.

Describe any plans for the participants in the currently proposed study to be re-contacted or recruited for future follow-up studies. (Note that participants should be informed of this potential for re-recruitment or future follow-up studies during the current study's consent process.)

None.

Please note the following KPNC IRB guidelines:

- Contact of prospective subjects will be limited to three (3) attempts in one week, for no longer than three (3) continuous weeks.
- Messages left will be limited to one (1) per week for no more than three (3) weeks.
- No more than two (2) recruitment mailings (email, flyer, brochure, etc.).
- If permission from the patient's PCP is necessary to contact the patient:
  - If PCP does not respond to request, recruitment attempts (phone, mailings, etc.) may not begin until 2 weeks after the PCP's permission was requested.
  - If PCP grants permission, recruitment attempts may begin with no waiting period.

**7.** Informed Consent Process

a. Written Consent

Describe how you will obtain and document consent, including:

**Phase 2**

A waiver of written informed consent is requested.

- Where, when and how the consent process will take place.

  N/A

- How the research team will ensure that subjects have sufficient time to consider whether to participate in the research

  N/A

- A process to ensure ongoing consent.

  N/A

- Steps that will be taken to minimize the possibility of coercion or undue influence.

  N/A

- Any steps that will be taken to ensure the subjects' understanding.

  N/A

- If you are planning to conduct remote informed consent, please describe the process in detail, including any electronic platforms. Indicate how the consent process will be documented.

  N/A

NOTE: For each federally-supported clinical trial, one IRB-approved informed consent form used to enroll subjects must be posted by the awardee or sponsor conducting the trial on a publicly available Federal website that will be established as a repository for such informed consent forms.

b. Waiver of Informed Consent

Provide rationale and justification for the Waiver of Informed Consent for this study, including:

- Explain how the proposed research presents no more than minimal risk to the study participants.

**Phase 2**

Study participants will receive augmented usual care from the HC APM (whose goal is to fully implement the new standard of care protocol with the intention of reducing the risk of hypoglycemia and improving safety). The attention presents no more than minimal risk to participants. (Data-only patients are not research subjects; they will receive usual care from their regular APM; the study will have no direct contact with the data-only patients.)

- Explain how the waiver of informed consent will not adversely affect the rights and welfare of the participants.

**Phase 2**

Receiving usual care with proactive attention from the HC APM will not affect the rights and welfare of participants.

- Explain why this research cannot practically be carried out without a waiver of informed consent. Note: research regulations require that justification for a waiver of consent explain why it is impracticable to perform the research, and not just impracticable to obtain consent. Practicability cannot be determined solely by considerations of convenience/cost/speed.

**Phase 2**

Receipt of usual care does not require obtaining consent at every step of every encounter. To introduce a consent requirement into the APM encounter would be inappropriate.

- If the research involves using identifiable private information or identifiable biospecimens, provide justification for why the research cannot practicably be carried out using deidentified information.

**Phase 2 )** The research could not be practicably carried out using de-identified information or data because linking the data extracted from the sources requires a common identifier to link the data.

- Assess whether it is appropriate to provide the subjects with additional pertinent information after participation.

**Phase 2**

In the course of usual care, patients are provided with pertinent information about their care and care options.

c. Waiver of Signed (Documented) Informed Consent

Provide rationale and justification for the Waiver of Signed (Documented) Informed Consent by identifying which of these three conditions applies and justification for how the criteria is met.

1) The research involves no more than minimal risk to participants AND involves no procedures for which written consent is normally required outside of the research context.

**Phase 2**

Participants will receive augmented usual care from the HC APM (whose goal is to fully implement the new standard of care protocol) which does not normally require written consent.

2) The signed consent form would be the only record linking the participants to the research, and the principal risk to participants would be potential harm resulting from a breach of confidentiality.

N/A

3) The subjects or legally authorized representatives are members of a distinct cultural group or community in which signing forms is not the norm.
    a. Describe the distinct cultural group or community
    b. Describe why signing forms is not the norm
    c. Explain how the research presents no more than minimal risk of harm to these subjects
    d. Provide an appropriate alternative mechanism for documenting that informed consent is obtained

N/A

d. Alteration of Informed Consent

Identify the required elements of informed consent that you wish to remove or alter.

N/A

Provide justification for their removal or alteration.

N/A

e. Non-English-Speaking Subjects

If subjects who do not speak English will be enrolled, describe how the consent discussion will take place and indicate if translated consent forms or short forms will be used. Confirm that an interpreter will assist with the initial consent process and subsequent study visits.

All contact with non-English-Speaking patients will utilize interpreters, in accordance with the usual clinical workflows when care is provided to these patients. As previously noted, a waiver of signed consent is requested, since this is not different from usual clinical care.

IMPORTANT NOTE: Please be aware that if it is expected that you will enroll non-English speakers in the study, short forms should not be used as the only Consent option for these individuals, they should only be used if a non-English speaking participant is unexpectedly encountered. However, the possibility of encountering non-English speaking potential subjects in the Bay Area is a possibility and this possibility should be considered, and budgeted for, when preparing the initial study submission for IRB review.

f. Assent of Children and Parent Permission

IMPORTANT NOTE: Child Consent may be obtained in certain situations (for example, conducting family planning or sexually transmitted disease (STD) research). In addition, for older children ages 16 and up who participate in an adult study, the consent document can be used in place of the assent document.

Describe how you will obtain and document assent/parental permission, including:

- Describe your plan for obtaining parent permission. The permission of one parent is generally sufficient for minimal risk research, or for greater than minimal risk research if there is the potential for direct benefit to the child. For studies involving greater than minimal risk with no prospect of direct benefit to the child, permission of both parents is required unless one parent is deceased, unknown, incompetent, or not reasonably available, or when only one parent has legal responsibility for the care and custody of the child.

  N/A Children will not be enrolled.

- Describe whether permission will be obtained from individuals other than parents, and if so, who will be allowed to provide permission.

  N/A

- Indicate whether assent will be obtained and documented from all, some, or none of the children.

  N/A

- If assent will only be obtained from some children (because of very young age, severe cognitive impairment, etc.), indicate which children will be required to assent and which will not.

  N/A

- When assent of children is obtained, describe whether and how it will be documented.

  N/A

- When subjects might reach the age of majority during the study, describe the plan to obtain consent from these subjects at that time using an adult consent form.

  N/A

g. Secondary Research for Which Consent is Not Required:
Note: Research involving the use of identifiable biospecimens does not apply to this section.
Note: Although consent may not be required, justification for a waiver of HIPAA Privacy Authorization may still be required.

Secondary Research of identifiable private information or identifiable biospecimens may not require informed consent if at least one of the criteria listed below is met. Select at least one of the following criteria and provide a rationale for how the criteria is met:

- Use of publicly available identifiable private information or identifiable biospecimens.

  N/A

- The information and/or biospecimens are recorded by the investigator in such a way that the identity of the subjects cannot be readily ascertained, and the investigator will neither contact the subjects nor re-identify subjects.

  N/A

- The research involves only information collection (i.e., no biospecimen collection) and the analysis of this identifiable health information is regulated by HIPAA.

  Data will be collected on data-only matched controls (receiving usual care subsequent to the regionwide dissemination of HOAP).

**8. HIPAA Privacy Rule Authorization**

a. Written HIPAA Privacy Rule Authorization:
Describe the plan to obtain a signed Privacy Rule Authorization from each subject.

**Phase 2**

A waiver of HIPAA authorization is requested.

b. Waiver of HIPAA Privacy Rule Authorization
If you will not obtain a signed HIPAA Privacy Rule Authorization or if you want to eliminate any required language from the authorization, provide the following rationale and justification.

- Explain why the research could not practicably be conducted without the waiver. Note: research regulations require that justification for a waiver of HIPAA Authorization explain why it is impracticable to perform the *research*, and not just impracticable to obtain HIPAA Authorization. Practicability cannot be determined solely by considerations of convenience/cost/speed.

  Receipt of usual care does not require obtaining HIPAA Privacy Rule Authorization at every step of every encounter. To introduce a consent requirement into the APM encounter would be inappropriate.

- Explain why access to and use of the PHI is necessary for the research.

It is necessary to access PHI to assemble the cohort of study participants and data-only patients. Without access to and use of the necessary PHI the research cannot be conducted. Direct contact with study participants is necessary for usual care as well as for this activity.

- Explain why the use or disclosure of PHI for the research poses no more than minimal risk to the subjects' privacy.

  Use of PHI poses no more than minimal risk as this study will store PHI in password protected or encrypted electronic files and stored on protected KP servers within each region and within KP networks secured by firewalls. Passwords will not be disclosed to any party not involved in the study, including study teams outside of the KP NCAL region. Patient identifiers will not appear in any part of the publications. When it is necessary to share PHI information among study parties within each respective KP region, password-protected/encrypted files will be transmitted through encrypted KP email or secure file transfer. For all these reasons the use and disclosure of PHI poses no more than minimal risk to the subjects' privacy.

- Provide an adequate plan to protect the PHI from improper use or disclosure.

  This study will store PHI in password protected or encrypted electronic files and stored on protected KP servers within KP network secured by firewalls. Passwords will not be disclosed to any party not involved in the study outside of each region. Patient identifiers will not appear in any part of the publications. When it is necessary to share PHI information among study parties within the immediate KP regional team, password-protected/encrypted files will be transmitted through encrypted KP email or secure file transfer. Only designated trained study staff within each regional study team will have access to all data collected as part of this study.

- Provide a plan to destroy identifiers at the earliest opportunity consistent with the purpose of the research.

  Personal identifiers associated with participants will be destroyed as soon as possible after the study has finished and after publication of all manuscripts. Since manuscript submission and revision process often requires re-access of patient information, patient identifiers will be kept until all publications generated from the study have been finished, at which time all patient identifiers will be destroyed at the close of the study.

c. **HIPAA Disclosure Accounting**

The Health Insurance Portability and Accountability Act (HIPAA) Privacy Rule gives patients the right to receive a listing, known as an accounting of disclosure, of their information that is disclosed to others for reasons other than treatment, payment, or health care operations. KP must account for all known disclosures of protected health information for research purposes without the individual's authorization (a waiver or alteration of HIPAA Authorization) both within a KP region (between a Permanente Medical Group, Kaiser Foundation Hospitals, and a Kaiser Foundation Health Plan) and outside of KP.

- What type of PHI is being disclosed?
  ☒ Clinical/Diagnostic
  ☒ Demographics
  ☒ Healthcare

- How many participants are expected to be enrolled in this study at your region?
  ☐ 49 or less individuals

  The investigator should maintain an individual accounting record of all Disclosures made by the research team that are subject to the HIPAA tracking requirements. The investigator must also transmit this tracking information within 20 days of Disclosure to the Regional Compliance Officer. Using: Health Connect Quick Disclosure or Complete the Disclosure of PHI about a single individual for a research purpose Form.

  ☒ 50 or more individuals

  If 50 or more individuals, please provide the information for each entity that sponsored the research where PHI is being disclosed. *Please note: If you are disclosing PHI to more than one entity, the following is needed for each entity.*

  - **Name**
    KPNC Region

  - **Address**
    2000 Broadway, Oakland, CA 94612

  - **Phone Number**
    510-891-3400

If disclosing PHI to more than one entity, please continue entering the required information below:
PHI will not be disclosed outside of the KP NCAL entity.

9. **Study Procedures**
   a. Description

Describe and explain the study design, including:

- A detailed chronological description of all research procedures.

**Phase 1:**

Aim 1

Months 1-4: Meetings with stakeholders will be convened by PIs.
Months 4-6: Finalize and disseminate “Hypoglycemia on a Page” (HOAP) (such as dissemination at all regional meetings with APMs, “steal this talk” presentations for PCPs, and electronic posting in the Clinical Library).

Aim 2

Months 2-4: Workflow/clinical pathway will be developed by PIs.

**Phase 2**

Aim 3

Months 5-6:

- Develop procedures and metrics for outcome evaluation
- Identify eligible patients (N=200)(have a high hypoglycemia risk score or a recent ED visit or hospitalization for hypoglycemia); randomly select study participants and data-only patients (matched sample)
- Collect baseline EMR data on study participants and data-only patients

Months 7-12:

- HC APM conducts outreach to study participants for assessment and evaluation; provides hypoglycemia intervention to study participants.
- 

Months 13-21

- Collect outcome data from EMR for study participants and data-only patients.

Months 21-24

- Analyze data and draft manuscript

- Procedures to monitor subjects for safety, including who will review the data and at what frequency for safety issues.

**Phase 2**

APM will routinely monitor study participants for safety and report any concerns to the Investigator.

- Procedures performed to lessen the probability or magnitude of risks.

**Phase 2**

Usual care has no more than minimal risk.

- The source records that will be used to collect information about subjects. (Attach all surveys, scripts, and data collection forms.)

**Phase 2**

Data will be collected from EMR.

- What information and/or biospecimens will be collected including during long-term follow-up.
  *Please note, if biospecimens being collected are being used to develop an investigational in vitro diagnostic device, an IDE for the device may be needed. Please review the IVD Device FAQs in IRBNet for guidance.*

**Phase 2**

Data collected from study participants and data-only patients

- Prescribing orders and pharmacy dispensing of medications including
- Durable medical equipment orders and dispensing of continuous glucose monitor devices and supplies
- Relevant lab results (e.g., HbA1c)
- ED visits or hospital admissions for hypoglycemia
- Hypoglycemia on the problem list
- Visit notes

- The duration of an individual subject's participation in the study.

**Phase 2**

Participation in the study will end within 12 months after enrollment of first participant and no later than 12/31/2024.

- The duration anticipated to enroll all study subjects.

Enrollment is expected to end by 01/31/2024.

- The estimated date for the investigators to complete this study (complete primary analyses) NOTE: It should be clear exactly which procedures will be conducted for the research as opposed to procedures the subjects would undergo (in the exact manner described in the protocol) even if they were not participating in the study.

The study is expected to be completed by 12/31/2024.

- Describe procedures that will be followed when subjects withdraw from the research, including withdrawal from intervention but continued information and/or biospecimen collection.

Participants who become non-adherent to care provided by HC APM will be followed up per the intent to treat analysis plan.

- Describe any anticipated circumstances under which subjects could be withdrawn from the research without their consent.

Discontinuation of Kaiser membership.

- Describe any procedures for orderly termination.

N/A

- If the study involves genetic testing or collection of genetic information, describe this.

N/A

- Clarify whether research involving biospecimens will (if known) or might include whole genome sequencing (i.e., sequencing of a human germline or somatic specimen with the intent to generate the genome or exome sequence of that specimen).

  N/A

- Does the study include basic and clinical research involving recombinant or synthetic nucleic acid molecules, including the creation and use of organisms and viruses containing recombinant or synthetic nucleic acid molecules, and is subject to NIH guidelines (Guidelines: https://osp.od.nih.gov/biotechnology/nih-guidelines/ and FAQs: https://osp.od.nih.gov/biotechnology/faqs-on-ibc-administration/)?

  ☐ Yes
  If yes, your study may require Institutional Biosafety Committee Review. Please contact kpnc.irb@kp.org for guidance.

  ☒ No

  Optional comments:
  N/A

b. Data Analysis

Describe the data analysis plan, including:

- Statistical procedures.

**Phase 2**

Statistical Design: The primary analysis for Aim 3 will be an intent-to-treat analysis using chi square tests and binomial regression. For the primary outcome, differences in the proportion of patients prescribed safer diabetes regimens (i.e., discontinuation of sulfonylureas, mealtime or mixed insulin) will be assessed at 6 months in the treatment vs. control arms. For the secondary outcomes, differences will be assessed in the proportion of patients who had an ED visit or hospitalization for hypoglycemia within 6 months, were prescribed CGM or prescribed glucagon, and were in glycemic control (HbA1c<8%) between the 2 study arms. The investigators will perform exploratory Heterogeneity of Treatment Effect (HTE) analyses for age, race and enrollment criterion (see above) by testing for statistical interactions with the exposure (treatment arm).

- When applicable, the power analysis.

**Phase 2**

Power Calculation: In preparation for this proposal, baseline use of hypoglycemia-prone medications (e.g., sulfonylureas, mealtime, or mixed insulin) was evaluated in 3921 KPNC patients identified as "high risk" by the KPNC hypoglycemia risk tool. Among these patients, 75% were on hypoglycemia-prone treatment. Approximately 60% of the intervention arm and 20% of the usual care arm will be prescribed safer diabetes regimens within 6 months compared

to baseline. The minimum number of subjects needed to detect this 40% absolute difference, assuming 90% power and a 10% attrition rate, is 86 patients (43 per arm). With an enrollment size of 200 (100 per arm), this trial is more than adequately powered to detect the hypothesized difference.

- Any procedures that will be used for quality control of collected data.

Data analysts routinely inspect the data for corrupt fields or extreme outliers.

c. Sharing of Results with Subjects

Describe whether results (study results or individual subject results, such as results of standard or research lab tests and genetic tests) will be shared with subjects or their providers and under which circumstances.

N/A

If the study carries a risk of incidental findings, describe your plan for evaluating these and determining whether and how subjects or their providers will be given this information.
N/A

If laboratory results will be shared with subjects or their healthcare providers, verify that the laboratory conducting the test is Clinical Laboratory Improvement Amendments (CLIA) certified.
N/A

Describe how study participants will be updated on research progress via newsletter, summary, etc. Consider incorporating such updates to study participants as part of ongoing retention efforts.
N./A

**10. Privacy, Confidentiality and Data Security**

Describe the steps that will be taken to protect subjects' privacy during recruitment, consent and study procedures.

Usual care procedures for protecting patient privacy will be used.

Describe the plan for storage of data and/or biospecimens including:

- Who will have access?

The PI, Co-I's and study team will have access to identifiable information from the medical record and other clinical databases. PHI will not be disclosed outside of study team.

- Where the data/materials will be stored and for how long. Indicate if data will be encrypted and password protected and if transportable/removable media will be used. Indicate if data will be stored on non-KP devices or sponsor-provided devices.

Data will be stored in password protected or encrypted electronic files and stored on protected KPNC servers within KP network secured by firewalls. Passwords will not be disclosed to any party

not involved in the study. Patient identifiers will not appear in any part of the publications. When it is necessary to share PHI information among study parties within KPNC, password-protected/encrypted files will be transmitted through encrypted KPNC email or secure file transfer. The information will be stored for the length of the project and destroyed after the final manuscripts are published. Data will be NOT stored on non-KP devices or sponsor-provided devices.

- If applicable, how will data be transmitted? (e.g. Encrypted email, secure file transfer, sponsor provided site)

Data will be transmitted via password-protected/encrypted files through encrypted KPNC email or secure file transfer.

- What identifiers will be included.

  MRNs, dates

- Any other steps that will be taken to ensure security (e.g., training of staff, authorization of access, password protection, encryption, physical security, and separation of identifiers from data and specimens, certificates of confidentiality).

  None, the investigators believe that the plan to protect the data is appropriately mitigated.

- Describe the plan to destroy/archive or retain data at the end of the study. If storing information and/or identifiable biospecimens for future research, complete the next section.

  Personal identifiers associated with participants will be destroyed as soon as possible after the study has finished and after publication of all manuscripts. Since manuscript submission and revision process often requires re-access of patient information, patient identifiers will be kept until all publications generated from the study have been finished, at which time all patient identifiers will be destroyed at the close of the study.

## 11. Information and/or Biospecimen Banking for Future Research

If you are creating a repository, please submit a separate protocol.

Indicate if biospecimens may be used for future research and whether that may include genetic research.

N/A

State if information or biospecimens will be sent to a separate repository. If data or specimens will be banked in a repository for future use as part of this protocol submission address the following questions:

- What will be banked and what identifiers will be associated with the information or biospecimens?

N/A

- Where and how will the information or specimens be stored?

  N/A

- For what purpose will the information or specimens be used? Include a general description of the types of research that may be conducted with the identifiable private information/biospecimens (e.g. research on cancer).

  N/A

- How will the information or specimens be accessed, and who will have access?

  N/A

- Describe the procedures to release information or specimens, including the process to request a release, approvals required for release, who can obtain information or specimens, and the information to be provided with specimens.

  N/A

- How long will identifiable private information/biospecimens be stored?

  N/A

**12. Collection of data from subjects electronically**

If you will collect any data from participants electronically (including email, website, etc.), explain:

- Does the study involve a Mobile Device or Application?  If yes, please indicate the source/manufacturer/developer of the product:

  No.

- How and what data will be collected.  Indicate if PHI is collected electronically (including via an app) and if there is any interface with the subject's personal accounts (for example: if subjects need to create an account to use the device, or if the device interfaces with an external source of data, or medical record).

  N/A

- How the information will be secured/transmitted/stored (encryption, password protection, etc.; may require consultation with IT department). Include a data flow diagram if data will flow through multiple parties (such as a hosting provider or coordinating center).

  N/A

- Any risks to the participants' privacy posed by using these methods (describe in consent, as applicable).

  N/A

- Describe if email containing PHI will be used to communicate with participants? (Per KP policy, securing messaging must be used for all communications containing PHI.)

  N/A

- How you will verify the participant's identity.

  N/A

**13. Disclosure of PHI to a collaborator**

If any data will be sent outside of this site, list each recipient (may list by role or category if the information is the same for several different entities). For each recipient, describe:

- The name and location of the individual/entity receiving the information.

  N/A

- What information and/or biospecimens will be sent.
  N/A

- Whether the information will be fully identifiable (PHI, if health information), a Limited Data Set, de-identified, or aggregate.

  N/A

- How the data/materials will be transferred securely (for instance, Secure File Transfer). Indicate if hard copy PHI will be stored/sent to a collaborator.

  N/A

- When applicable, clarify whether there are written assurances from collaborators that PHI will not be reused or re-disclosed to any other entity. Describe assurances that PHI will be stored securely. Describe mechanisms (e.g., intrusion detection software or regular electronic system activity audits or monitoring) for determining if KP PHI has been inappropriately or illegally accessed, used, disclosed, or modified.

  N/A

**14. Provisions to Monitor Data to Ensure the Safety of Subjects**

This is required when research involves more than Minimal Risk to subjects.

The plan might include establishing a data monitoring committee and a plan for reporting data monitoring committee findings to the IRB and the sponsor. Describe:

- Who will monitor the study data for safety?

  N/A. This study involves no more than minimal risk to subjects.

- The plan to periodically evaluate the data collected regarding both harms and benefits to determine whether subjects remain safe.

  N/A

- What data are reviewed, including safety data, untoward events, and efficacy data.

  N/A

- How the safety information will be collected (e.g., with case report forms, at study visits, by telephone calls with participants).

  N/A

- The frequency of data collection, including when safety data collection starts.

  N/A

- The frequency or periodicity of review of cumulative data.

  N/A

- Criteria for taking action on monitoring findings (for instance, stopping rules, immediate suspension, reporting, protocol changes, changes to monitoring frequency or plan).

  N/A

- For studies monitored by a DSMB/C, describe the committee membership and structure, meeting format, and quorum requirements.  Upload the board/committee charter, if one exists.

  N/A

**15. Risks and Benefits**

a. Risks to Subjects

List the reasonably foreseeable risks, discomforts, hazards, or inconveniences to the subjects related the subjects' participation in the research. Consider physical, psychological, social, legal, and economic risks.

No foreseeable risks, discomforts, hazards, or inconveniences to participants are anticipated.

Describe the probability, magnitude, duration, and reversibility of the risks.
N/A

If applicable, indicate which procedures may have risks to the subjects that are currently unforeseeable.

N/A

If applicable, indicate which procedures may have risks to an embryo or fetus should the subject be or become pregnant.

N/A

If applicable, describe risks to others who are not subjects and risks to Kaiser Permanente

N/A

b. Potential Benefits to Subjects
Describe the potential benefits that individual subjects may experience from taking part in the research. Include as may be useful for the IRB's consideration, the probability, magnitude, and duration of the potential benefits. Indicate if there is no direct benefit. Do not include benefits to society or others.

The study is intended to reduce risk of hypoglycemia and improve patient safety. Participants may experience these benefits.

c. Risks to KP
Is there anything about the nature of this study which, if revealed to the public, could put KP at risk or competitive disadvantage? ☐ **Yes** ☒ **No**
If "yes" describe in detail.

**16. Economic Burden to Subjects**
Describe any costs that subjects may be responsible for because of participation in the research study (for example, co-pays; paying for treatment, therapies, or other interventions, or the delivery of these) and how you will inform participants of these costs prior to their enrollment in this study.

None

**17. Compensation to Participants**
Describe any compensation provided to participants, for example, for time inconvenience, discomfort, travel, or in the event of research related injury. If applicable, describe how you will inform participants of this prior to their enrollment in the study, including if payment will be prorated if the subject withdraws early from the study.

NOTE: payment may not be withheld as an incentive for participants to complete any portion of the study.

None.

**18. Resources Available**
Describe any special resources or expertise required to conduct the study.

Click or tap here to enter text.

**19. Principal Investigator**

a. Has the Principal Investigator (PI) previously been approved as PI for a study within KPNC? ☒ Yes ☐ No

b. Has the PI been audited/assessed within the past three years? ☐ Yes ☒ No
If Yes, check all that apply and provide the outcome/findings:
☐ FDA ☐ RQCC ☐ CTP ☐ CCRU
Outcome/Findings: Click or tap here to enter text.

c. Is the PI currently under a Corrective And Preventative Action (CAPA) plan?
☐ Yes ☒ No
If Yes, provide a brief description: Click or tap here to enter text.

**20. Required Approvals**

a. Describe any approvals that will be obtained prior to commencing the research. (e.g., school, external site. funding agency, or other KP departments). Be sure to list each KP site (for example, KP San Francisco Hospital, Division of Research, KP Oakland Reg – 1800 Harrison, etc.)

Division of Research

b. **Facility-Based Research:** KPNC IRB requires that the Principal Investigator (PI) obtain approval signatures at **each KPNC facility** where research activity will occur. These may include local research chair, Physician-in-Chief, departmental chair, Area Manager, information technology review (as appropriate), and Division of Research scientific review (student research).

List each KP facility that research activity will occur and specify whose signature has been/will be obtained.

Name of Facility: SSF

Local Research Chair (LRC): Robert Li

Chief of Service: Joanie Loh, MD

Physician-in-chief (PIC): John Skerry, MD

Area Managers (if applicable): Sheila Gilson

c. **Non-Facility-Based Research** (ex: Northern California Regional and Program Office): For non-facility-based research, the PI's supervisor and department head are required in lieu of approvals from the Chief of Service, Physician-in-chief, and Area Manager. The Central Research Committee Chairperson serves as the LRC. As such, list each KP non-facility that research activity will occur and confirm whose signature has been/will be obtained.

PI's Supervisor: Tracy Lieu, MD (Richard Grant)

Department Head: ?

Central Research Committee Chairperson: Michael Silverberg, MD

d. **Sites with no Local Research Chair:** For sites with no Local Research Chair, the Central Research Committee Chairperson serves as the Local Research Chair:

Site: N/A

Central Research Committee Chairperson: N/A

**21. Drugs or Devices**

NOTE: see the ICH-GCP guidance for a summary of investigator and sponsor responsibilities in clinical trials.

List all drugs and devices used in the research and the purpose of their use, and their regulatory approval status.

N/A

a. Drug Studies

If the research involves drugs and is investigator-initiated, indicate whether there is any possibility that the results will be reported to FDA (e.g. as part of a new drug application [NDA]).

N/A

If the drug is investigational (has an IND), confirm that you will comply with all applicable FDA requirements for investigators.

N/A

Confirm that you will follow applicable KP pharmacy policies and procedures.

N/A

Describe your plan for drug storage, handling, and accountability, including distribution, return, and destruction of the drug(s).

N/A

b. Device Studies:

If this is a device study and you think the device is Non-Significant Risk, include justification here or upload it as a separate document along with any available device information (instructions for use, etc.).

N/A

If the research involves devices and is investigator-initiated, indicate whether there is any possibility that the results will be reported to FDA (e.g. as part of a premarket approval application [PMA]).

N/A

If the device has an IDE or a claim of abbreviated IDE (Non-Significant Risk device), confirm that you will comply with all applicable FDA requirements for investigators.

N/A

Describe the device, the manufacturing process, and the device labeling, including safety instructions or warnings. If available, this may be addressed in separately uploaded device information (such as instructions for use).

N/A

Describe device storage, handling, and accountability, including how access to the device will be limited to appropriate personnel and how you will ensure the device will be used only for appropriate study subjects.

N/A

**22. Multi-Site Research**

a. If this is a multi-site study and you are the lead investigator or this site will be the coordinating center for any activity, describe the processes to ensure communication among sites, such as:

- All sites have the most current version of the protocol, consent document, and HIPAA authorization.

  *N/A*

- All required approvals have been obtained at each site (including approval by the site's IRB of record).

  *N/A*

- All modifications have been communicated to sites, and approved (including approval by the site's IRB of record) before the modification is implemented.

  *N/A*

- All engaged participating sites will safeguard data as required by local information security policies.

  *N/A*

- All local site investigators conduct the study appropriately.

*N/A*

b. Describe the method for communicating to engaged participating sites the following:

- Problems.

  N/A

- Interim results.

  N/A

- The closure of a study.

  N/A

c. Describe any special resources or expertise required to conduct the study.

*N/A*

**23. Community-Based Participatory Research**
Describe involvement of the community in the design and conduct of the research.

N/A

Describe your plan for ensuring that community research partners are appropriately trained in human subjects' protection.

N/A

NOTE: "Community-based Participatory Research" is a collaborative approach to research that equitably involves all partners in the research process and recognizes the unique strengths that each brings. Community-based Participatory Research begins with a research topic of importance to the community, has the aim of combining knowledge with action and achieving social change to improve health outcomes and eliminate health disparities.

**INSTRUCTIONS:**

- Please ensure you are using the most recent version available in IRBNet.
- Complete this Protocol Template only when there is **no** existing authored protocol provided for this study.
- If you are conducting a data-only study with no prospective or interventional components, use the Data Only Protocol Template instead.
- This Protocol Template is to be used in conjunction with the SMART KP IRB Core Data Form.
- Enter your responses to each question directly below the BLUE text in the fillable field.
- When completing this Protocol Template, if a section does not apply to your study then enter "N/A."

1. **Protocol**

Protocol Title

**Testing a new population management model for hypoglycemia prevention in high-risk KPNC members.**

Principal Investigator

Lisa Gilliam, MD, PhD
Richard Grant, MD, MPH

Version Date

January 27, 2025

Form Author

Andrew Karter

2. **Objectives**

Describe in plain language the purpose, specific aims, or objectives and indicate the primary goal(s) of the study (e.g. safety, tolerability, effectiveness, feasibility, pilot study, etc.). State the hypotheses to be tested. State primary and any secondary study endpoints.

The primary research question is whether using a "Hypoglycemia Champion" (HC) to target high risk patients with type 2 diabetes (T2D) is a feasible and effective model of care to reduce hypoglycemia risk in this population. The HC will be a specially trained accountable population manager (APM) who will implement a new clinical guideline intended to reduce risk of hypoglycemia.

The aims of this study are to:

1. Convene meetings with stakeholders (endocrinologists within and outside of KP) to develop an evidence-based, expert consensus clinical guideline, "Hypoglycemia on a Page" (HOAP), for hypoglycemia prevention in patients with T2D at high risk of hypoglycemia; publish HOAP in the KPNC clinical library and disseminate for general use by KPNC diabetes care providers (APMs and PCPs) via regional presentations as the new standard of care;
2. Develop a workflow/clinical pathway for use by a "Hypoglycemia Champion" (HC), a clinical pharmacist/accountable population manager (APM) ("HC APM") who will proactively outreach and

apply the HOAP guideline in the management of KPNC members identified as having high hypoglycemic risk;

3. Conduct an evaluation of whether usual care augmented by HC APM (augmented usual care) is associated with better diabetes regimen safety among KPNC members at high risk of hypoglycemia compared to patients receiving usual care. The study hypothesis is that at 6 months, eligible members assigned to the HC APM (augmented usual care) will be prescribed safer diabetes regimens compared to usual care (control). Care for all eligible members (usual care and augmented usual care) will include available use of the HOAP guidelines.

## 3. Background

a. Scientific Background

Provide the scientific or scholarly background for, rationale for, and significance of the research based on the existing literature and how will it add to existing knowledge. A list of references or bibliography must be included as part of this document or uploaded separately.

Hypoglycemia in patients with type 2 diabetes (T2D) is a significant adverse drug event associated with the hypoglycemia-inducing diabetes medications, insulin and sulfonylureas. Hypoglycemia has been poorly addressed by health plans and all but ignored by national performance metrics such as HEDIS.

Hypoglycemia is common (in a survey of 20,188 diabetes patients from KPNC, 12% reported having ≥1 severe hypoglycemia events requiring third-party assistance in the previous 12 months), morbid (associated with falls, fall-related fractures, automobile accidents, cardiovascular events, dementia, and even death), and costly (total annual direct medical costs ~$1.8 billion in 2009 in the US). Hypoglycemia often leads to significant diabetes distress and nonadherence to prescribed diabetes medications. Providers are challenged to address the competing demands of prescribing glucose-lowering medications to meet glycemic targets while avoiding hypoglycemia.

In addition, there are now strategic considerations for reducing hypoglycemia risk. NCQA is proposing a new HEDIS measure assessing emergency department visits for hypoglycemia in older adults with diabetes for measurement year 2023 (MY2023). There is little time to prepare before its proposed implementation in 2023. Unlike most of the existing HEDIS measures, this new measure is focused on patients >65 years of age and is not capped at 75 years of age, which matches the age range where hypoglycemia risk is highest. Hypoglycemia risk can be mitigated, but there exist no well-validated, population management approaches for hypoglycemia prevention. Our proposal intends to address this gap by developing and evaluating a practical population management approach to hypoglycemia prevention.

Severe hypoglycemia is the most common adverse drug effect associated with diabetes medications. However, hypoglycemia in patients with T2D is not adequately recognized, documented, or treated by care providers. Ever since landmark clinical trials (e.g., UKPDS) demonstrated that lowering blood glucose levels reduced the risk of long-term diabetes complications, the medical community has prioritized A1C reduction, often underestimating the risks of diabetes medications, especially hypoglycemia. Each year, approximately 11% of KPNC patients with type 2 diabetes experience severe hypoglycemia (a hypoglycemic episode requiring third-party assistance), with the risk increasing with age and duration of diabetes.(Karter, 2018) In older adults, approximately 13% of emergency department admissions for adverse drug events are due to hypoglycemia (primarily in insulin-treated

patients)(Shehab, 2016), and annual hospital admissions for hypoglycemia now surpass those for hyperglycemia.(Lipska, 2014)

The research question is whether an intervention by a hypoglycemia champion directed towards patients at high risk of hypoglycemia can reduce hypoglycemia events more than usual care (which will include a new treatment algorithm aimed at hypoglycemia risk reduction).

We previously developed a hypoglycemia risk tool which can stratify patients into low, intermediate or high risk for ED visit or hospitalization for hypoglycemia.(Karter, 2017; Karter, 2019) We demonstrated that patients classified as high risk for hypoglycemia were 35 times more likely to have an ED or hospital visit for hypoglycemia in the subsequent 12 months, compared to patients classified as low risk. However, it is important to note that ED or hospital visits for hypoglycemia are just the tip of the iceberg: 95% of patients experiencing severe hypoglycemia are cared for outside of the medical system (i.e., assisted by family or others).(Karter, 2018)

Currently, no well-validated, population management approach for hypoglycemia prevention exists. Front line clinicians need information and guidance on how to minimize hypoglycemia risk while optimizing glycemic control to reduce long-term diabetes complications. Thus, the development and evaluation of interventions to address hypoglycemia risk are needed and are highly relevant to TPMG care delivery, patient safety, and strategic goals of maintaining stellar HEDIS performance metrics.

This work has strategic implications for our organization because a new HEDIS measure proposed for measurement year 2023 will assess emergency department admissions for hypoglycemia in older adults with diabetes. Our proposal will address this gap in organizational clinical care by developing and evaluating a practical, proactive, population management approach to hypoglycemia prevention for members with T2D at high risk of hypoglycemia

b. <u>Preliminary Data</u>
Describe any relevant preliminary data.

None

4. **Study Design**
Describe the overall approach of the study (e.g. prospective, interventional, observational, retrospective, etc.). If your study includes more than one group, arm, or subject population, describe that here (for example, a study of both subjects and their caregivers, or a study with both a prospective interventional arm and a retrospective chart review arm).

**Phase 1 (not Human Subjects Research)**
In **Phase 1** of this study (Aims 1 & 2), the investigators will:

- convene meetings with endocrinologists to develop and publish a new hypoglycemia prevention guideline, "Hypoglycemia on a Page" (HOAP) [Aim 1], and
- develop a clinical workflow for a clinical pharmacist/APM Hypoglycemia Champion ("HC APM") to proactively apply the new guideline [Aim 2]

Aim 1 – Develop and disseminate "Hypoglycemia on a Page" (HOAP)

- Develop an evidence-based hypoglycemia prevention algorithm to improve the safety of medication regimens for patients with T2D. As with prior guidelines (e.g., "PHASE on a Page"), the investigators will convene regional and national stakeholders to review evidence, gather clinical insights, integrate feedback, and reach consensus for a single standard clinical care guideline for hypoglycemia prevention in high-risk members across KPNC.
- Disseminate HOAP regionally for use by KPNC diabetes care providers (APMs and PCPs)

Aim 2 – Develop the workflow protocol for use by the "Hypoglycemia Champion" (HC) APM, a clinical pharmacist who will be trained to apply the HOAP guideline to the proactive management of members at high risk of hypoglycemia. This structural change will be modeled after KP's highly effective PHASE cardiovascular risk management program.

**Phase 2**

In **Phase 2** of this study (Aim 3), the investigators will:

- conduct a prospective evaluation of whether proactive intervention by the Hypoglycemia Champion (augmented usual care) is associated with better diabetes regimen safety among KPNC members at high risk of hypoglycemia compared to usual care (control) [Aim 3].

Aim 3 –Conduct a prospective evaluation of whether proactive intervention by the Hypoglycemia Champion APM (i.e., assignment to augmented usual care group) is associated with better diabetes medication regimen safety among KPNC members at high risk of hypoglycemia compared to usual care (control).

- Randomly assign eligible patients to receive proactive intervention by the HC APM (augmented usual care group) for comparison to data-only patients receiving usual care. All eligible patients will be managed according to the regionally disseminated HOAP as new standard of care). All medical decisions for all patients (augmented usual care and usual care) are made by each member's medical care team responsible for his or her care (not by study personnel).

- Primary Outcome: Proportion of high-risk patients prescribed safer diabetes regimens (discontinuation at 6 months compared to pre-baseline of sulfonylureas, mealtime [rapid-/short-acting], or mixed insulin) comparing study participants (augmented usual care) to data-only patients (usual care controls). The study hypothesis is that more study participants will be prescribed safer diabetes regimens compared to data-only patients. Baseline pharmacy dispensing data and medication discontinuation orders during follow up will be collected directly from EHR/Clarity tables.
    i. As researchers, patients will not be contacted directly at any time.
    ii. All care will be delivered under approved protocols by KPNC clinicians.
    iii. All evaluation data related to clinical outcomes will be collected directly from the medical record of usual care.
- Secondary Outcomes: the investigators will also examine differences between study participants and data-only patients in glucagon prescribing, CGM use, hypoglycemia on the

patient's problem list, HbA1c<8% (HEDIS-defined glycemic control metric), and ED visits or hospitalizations for hypoglycemia based on primary diagnosis. The study hypothesis is that study participants will have more glucagon prescribing, CGM use, and inclusion of hypoglycemia on the problem list, with stable glycemic control (HbA1c<8%), and fewer ED visits or hospitalizations for hypoglycemia. The investigators will also conduct Heterogeneity of Treatment Effect (HTE) analyses by age (<75 vs. ≥ 75 years), enrollment criterion (hypoglycemia prediction algorithm vs ED/hospital hypoglycemia admission), and race/ethnicity.

Assess whether this study involves a clinical trial. Clinical trial means a research study in which one or more human subjects are prospectively assigned to one or more interventions (which may include placebo or other control) to evaluate the effects of the interventions on biomedical or behavioral health-related outcomes.

**Phase 2**
While not a clinical trial (since all patients will be offered usual care), Aim 3 will include a prospective evaluation of whether receiving proactive intervention by the Hypoglycemia Champion (augmented care delivered by an APM trained in the new standard of care) is associated with better diabetes regimen safety among KPNC members at high risk of hypoglycemia compared to usual care (usual care). All KPNC patients at high risk for hypoglycemia are expected to receive the new HOAP protocol delivered by their usual APM. Outcomes among study participants in Aim 3 will compared to data-only patients receiving usual care.

Describe whether the study involves educational tests, survey procedures, or interview procedures.

N/A

Describe whether the research involves benign behavioral interventions on **adult** subjects. Note: Benign behavioral interventions are brief in duration, harmless, painless, not physically invasive, not likely to have a significant adverse lasting impact on the subjects, and not offensive or embarrassing to the subjects. Examples include having subjects play online games or solving puzzles under various noise conditions.

N/A

If the study involves either educational tests, survey procedures, interview procedures, or benign behavioral interventions **(on adults)**, specify whether one of the following criteria is met:

(i) The information obtained is recorded by the investigator in such a manner that the identity of the human subjects cannot readily be ascertained, directly or through identifiers linked to the subjects

N/A

(ii) Any disclosure of the human subjects' responses outside the research would not reasonably place the subjects at risk of criminal or civil liability or be damaging to the subjects' financial standing, employability, educational advancement, or reputation

N/A

(iii) The information obtained is recorded by the investigator in such a manner that the identity of the human subjects can readily be ascertained, directly or through identifiers linked to the subjects, and an IRB conducts a limited IRB review to make the determination

N/A

**5. Study Population**

a. Number of Subjects

State the number of subjects you plan to include at the KP region to which this study is being submitted. If applicable, distinguish between the number of subjects who are expected to be enrolled/screened and the number of subjects needed to complete the research procedures (e.g. number of subjects excluding screen failures).

**Phase 1**

Aim 1 meeting participants are not research subjects. They are collaborators with subject matter expertise. Aim 2 is an activity with no research subjects.

**Phase 2 )**

Aim 3 will identify 200 eligible patients receiving usual care from KPNC; half of these will be randomly allocated to receive augmented usual care (i.e., additional oversight be a Hypoglycemia Champion APM).

As appropriate, differentiate between different populations of subjects within the same study (e.g. subject/caregiver, parent/child, patient/physician).

In this study, the only research subjects are study participants in Aim 3 receiving augmented usual care from the HC APM.

If this is a multicenter study, indicate the total number of subjects to be accrued across all sites.

N/A

If this study has a data only component, indicate the total number of patient records to be accessed.
Control subjects for Aim 3 will have 100 matched subjects for data-only.

b. Inclusion and Exclusion Criteria

- Describe the criteria that define who will be included or excluded in your final study sample.

**Phase 2**

Eligibility criteria for Aim 3 (study participants and data-only patients) will include patients with type 2 diabetes (T2D) at high risk for hypoglycemia, including those 1) designated as "high-risk" by the existing hypoglycemia risk stratification tool[1, 2] developed by the investigator team and currently implemented into Health Connect, or 2) who have had an ED visit or hospitalization for hypoglycemia (as primary or principal diagnosis, respectively) in 6 months prior to cohort baseline (date of enrollment in cohort).

No exclusion criteria.

- Describe how individuals will be screened for eligibility.

**Phase 2**

The data analyst will identify a cohort of 200 eligible patients from the KPNC Diabetes Registry based on the inclusion and exclusion criteria. 100 study participants will be randomly allocated to receive augmented usual care; the remaining 100 data-only patients will be selected for comparison. No screening is needed.

- If you are planning to conduct remote informed consent, how will you assure that participants should not be excluded from the study? e.g. how will you determine that potential participants can understand, adequately hear, have appropriate decision making capacity, and have a copy of the informed consent form?

**Phase 2**

A waiver of informed consent is requested.

- If information or biospecimens will be obtained for the purpose of screening, recruiting, or determining eligibility, informed consent is not required if one of the following criteria is met. Select one of the following criteria and provide a brief explain for how the criteria is met.

  ☒ The investigator will obtain information through oral or written communication with the prospective subject or legally authorized representative, or

  ☐ The investigator will obtain identifiable private information or identifiable biospecimens by accessing records or stored identifiable biospecimens

  N/A

  IMPORTANT NOTES: Although informed consent may not be required, HIPAA Privacy Authorization may still be required. Also, if this study is FDA-regulated, then consent may be required.

- Describe the plan for disposition of information/biospecimens collected during recruitment/screening in the event of a screen failure or when a potential subject is contacted but declines participation (e.g., destroyed immediately, destroyed at end of study, retained for separate analysis or so that subjects are not contacted repeatedly about participation after they have declined, etc.).

N/A

c. Subjects Vulnerable to Coercion or Undue Influence

Indicate whether you will include or exclude each of the following special populations. Justify the inclusion of any of these populations. Describe additional safeguards to protect the rights and welfare of these subjects. Note: This refers to subjects who are known members of these populations upon enrollment or at any time during the study.

- Children

  Study will not include children.

- Neonates of uncertain viability or nonviable neonates (up to 28 days post birth)

  N/A

- Prisoners (NOTE: The KP IRB does not have the appropriate membership to review research involving prisoners. Consultation with the IRB Office will be required.)

IMPORTANT NOTE: Consider whether subjects will be in a vulnerable category at the time of information/biospecimen collection or during analysis. For instance, if you collect information/biospecimens about children who were ages 12 – 15 from years 2000 – 2002, you know that now those individuals are no longer children.

Individuals with Impaired Decision-Making Capacity

Indicate how you will assess decision making capacity and cognitive function. This process must be tailored based on the risk and design of the study.

**Phase 2**

Decision-making capacity or cognitive function of participants will not be assessed.

State whether individuals with impaired decision-making capacity will be included.

N/A

Explain the extent of cognitive impairment (complete, fluctuating, progressive, or temporary).

N/A

Justify their inclusion and explain any protections to mitigate risk (such as the involvement of a caregiver or legally authorized representative).

N/A

Describe the process to determine whether an individual is capable of consent, and submit any documents that will be used assess decisional capacity.

N/A

List the individuals from whom permission will be obtained in order of priority. (E.g., durable power of attorney for health care, court appointed guardian for health care decisions, spouse, and adult child.

N/A

Describe the process for assent of the subjects by addressing the following:

- Whether assent will be required of all, some, or none of the subjects. If assent will be obtained from some subjects, indicate which subjects will be required to assent and which will not.

  N/A

- If assent will not be obtained from some or all subjects, an explanation of why not.

  N/A

- When assent is obtained, describe how it will be documented.

  N/A

HIV Status: N/A

If the study will be ascertaining subject HIV status for study exclusion/inclusion, please indicate how:

☐ Prospective laboratory HIV testing of subjects for the study

☐ Surveying subjects about their HIV status

☐ KPNC HIV Registry access or existing electronic health record data*

*If you checked this option, you must first gain approval from the HIV Steering Committee by contacting Michael J. Silverberg, PhD, MPH. The HIV Steering Committee approval must be submitted to IRBNet.

Will TPMG physicians be directly contacted to enroll as study participants? ☐ **Yes** ☒ **No**

**If "yes" the contact must be approved by Yi Fen Irene Chen, MD, Associate Executive Director, TPMG, prior to IRB review. Dr. Chen's approval must be submitted to IRBNet.**

Other Populations Targeted for Recruitment
If you are targeting a population that may be vulnerable to coercion or undue influence based on the specific circumstances of the study, describe how you will ensure that participation is voluntary and minimize any added risk. (Common examples include employees, students, economically or educationally disadvantaged persons, etc.)

N/A

d. Setting
Describe the sites or locations where your research team will conduct the research.

**Phase 1**
Aim 1: These small group meetings will be held virtually using a KP IT approved application.

Aim 2: The workflow will be developed under the direction of the PIs at SSF and DOR.

**Phase 2**

Aim 3: The HC APM will be based in SSF; data analysis will be conducted at the Division of Research.

If this is a multi-site study:

- Specify what procedures are being performed at this site or by this site's personnel (consider recruitment, consent process, study procedures, information/biospecimen analysis, etc.).

  N/A

- State how each site will satisfy its IRB review requirements. Indicate if you are asking this site's IRB to rely on another IRB or if another institution would like to rely on this site's IRB and include this information in the eIRB Initial Project submission

  N/A

For research conducted outside this site describe: (Community, Reservations etc.)

- Describe site-specific regulations or customs affecting the research at that location.

  N/A

- Local scientific and ethical review structure outside this site.

  N/A

**6.** Recruitment Methods

Describe how study participants will be recruited and enrolled.

**Phase 2**

There will be no recruitment. All patients will already be receiving usual care from an APM.

- For study participants, the HC will communicate with each participant's provider (usual APM or physician) to coordinate care; the study participant will receive proactive intervention by the HC as an augmentation to their usual care.
- For the data-only patients, there will be no contact.

Indicate whether you will openly recruit using advertisements, websites, flyer, or brochures. (Upload the final versions of all recruitment materials to your submission to the IRB.)

N/A

Indicate if you plan to do targeted recruitment using existing records or referral. Please submit final versions of all referral emails/scripts.

N/A

Describe, by position/title, who will be recruiting and enrolling participants (providing the specific names of research team members is not necessary).

**Phase 2**
Participants will be enrolled by the Hypoglycemia Champion who will be a pharmacist APM.

Describe any plans for the participants in the currently proposed study to be re-contacted or recruited for future follow-up studies. (Note that participants should be informed of this potential for re-recruitment or future follow-up studies during the current study's consent process.)

None.

Please note the following KPNC IRB guidelines:

- Contact of prospective subjects will be limited to three (3) attempts in one week, for no longer than three (3) continuous weeks.
- Messages left will be limited to one (1) per week for no more than three (3) weeks.
- No more than two (2) recruitment mailings (email, flyer, brochure, etc.).
- If permission from the patient's PCP is necessary to contact the patient:
  - If PCP does not respond to request, recruitment attempts (phone, mailings, etc.) may not begin until 2 weeks after the PCP's permission was requested.
  - If PCP grants permission, recruitment attempts may begin with no waiting period.

**7.** Informed Consent Process

a. Written Consent
Describe how you will obtain and document consent, including:

**Phase 2**
A waiver of written informed consent is requested.

- Where, when and how the consent process will take place.

  N/A

- How the research team will ensure that subjects have sufficient time to consider whether to participate in the research

  N/A

- A process to ensure ongoing consent.

  N/A

- Steps that will be taken to minimize the possibility of coercion or undue influence.

  N/A

- Any steps that will be taken to ensure the subjects' understanding.

  N/A

- If you are planning to conduct remote informed consent, please describe the process in detail, including any electronic platforms. Indicate how the consent process will be documented.

  N/A

NOTE: For each federally-supported clinical trial, one IRB-approved informed consent form used to enroll subjects must be posted by the awardee or sponsor conducting the trial on a publicly available Federal website that will be established as a repository for such informed consent forms.

b. Waiver of Informed Consent

Provide rationale and justification for the Waiver of Informed Consent for this study, including:

- Explain how the proposed research presents no more than minimal risk to the study participants.

**Phase 2**

Study participants will receive augmented usual care from the HC APM (whose goal is to fully implement the new standard of care protocol with the intention of reducing the risk of hypoglycemia and improving safety). The attention presents no more than minimal risk to participants. (Data-only patients are not research subjects; they will receive usual care from their regular APM; the study will have no direct contact with the data-only patients.)

- Explain how the waiver of informed consent will not adversely affect the rights and welfare of the participants.

**Phase 2**

Receiving usual care with proactive attention from the HC APM will not affect the rights and welfare of participants.

- Explain why this research cannot practically be carried out without a waiver of informed consent. Note: research regulations require that justification for a waiver of consent explain why it is impracticable to perform the research, and not just impracticable to obtain consent. Practicability cannot be determined solely by considerations of convenience/cost/speed.

**Phase 2**

Receipt of usual care does not require obtaining consent at every step of every encounter. To introduce a consent requirement into the APM encounter would be inappropriate.

- If the research involves using identifiable private information or identifiable biospecimens, provide justification for why the research cannot practicably be carried out using deidentified information.

**Phase 2 )** The research could not be practicably carried out using de-identified information or data because linking the data extracted from the sources requires a common identifier to link the data.

- Assess whether it is appropriate to provide the subjects with additional pertinent information after participation.

**Phase 2**

In the course of usual care, patients are provided with pertinent information about their care and care options.

c. Waiver of Signed (Documented) Informed Consent

Provide rationale and justification for the Waiver of Signed (Documented) Informed Consent by identifying which of these three conditions applies and justification for how the criteria is met.

1) The research involves no more than minimal risk to participants AND involves no procedures for which written consent is normally required outside of the research context.

**Phase 2**

Participants will receive augmented usual care from the HC APM (whose goal is to fully implement the new standard of care protocol) which does not normally require written consent.

2) The signed consent form would be the only record linking the participants to the research, and the principal risk to participants would be potential harm resulting from a breach of confidentiality.

N/A

3) The subjects or legally authorized representatives are members of a distinct cultural group or community in which signing forms is not the norm.
   a. Describe the distinct cultural group or community
   b. Describe why signing forms is not the norm
   c. Explain how the research presents no more than minimal risk of harm to these subjects
   d. Provide an appropriate alternative mechanism for documenting that informed consent is obtained

N/A

d. Alteration of Informed Consent

Identify the required elements of informed consent that you wish to remove or alter.

N/A

Provide justification for their removal or alteration.

N/A

e. Non-English-Speaking Subjects

If subjects who do not speak English will be enrolled, describe how the consent discussion will take place and indicate if translated consent forms or short forms will be used. Confirm that an interpreter will assist with the initial consent process and subsequent study visits.

All contact with non-English-Speaking patients will utilize interpreters, in accordance with the usual clinical workflows when care is provided to these patients. As previously noted, a waiver of signed consent is requested, since this is not different from usual clinical care.

IMPORTANT NOTE: Please be aware that if it is expected that you will enroll non-English speakers in the study, short forms should not be used as the only Consent option for these individuals, they should only be used if a non-English speaking participant is unexpectedly encountered. However, the possibility of encountering non-English speaking potential subjects in the Bay Area is a possibility and this possibility should be considered, and budgeted for, when preparing the initial study submission for IRB review.

f. Assent of Children and Parent Permission

IMPORTANT NOTE: Child Consent may be obtained in certain situations (for example, conducting family planning or sexually transmitted disease (STD) research). In addition, for older children ages 16 and up who participate in an adult study, the consent document can be used in place of the assent document.

Describe how you will obtain and document assent/parental permission, including:

- Describe your plan for obtaining parent permission. The permission of one parent is generally sufficient for minimal risk research, or for greater than minimal risk research if there is the potential for direct benefit to the child. For studies involving greater than minimal risk with no prospect of direct benefit to the child, permission of both parents is required unless one parent is deceased, unknown, incompetent, or not reasonably available, or when only one parent has legal responsibility for the care and custody of the child.

  N/A Children will not be enrolled.

- Describe whether permission will be obtained from individuals other than parents, and if so, who will be allowed to provide permission.

  N/A

- Indicate whether assent will be obtained and documented from all, some, or none of the children.

  N/A

- If assent will only be obtained from some children (because of very young age, severe cognitive impairment, etc.), indicate which children will be required to assent and which will not.

  N/A

- When assent of children is obtained, describe whether and how it will be documented.

  N/A

- When subjects might reach the age of majority during the study, describe the plan to obtain consent from these subjects at that time using an adult consent form.

  N/A

g. Secondary Research for Which Consent is Not Required:
Note: Research involving the use of identifiable biospecimens does not apply to this section.
Note: Although consent may not be required, justification for a waiver of HIPAA Privacy Authorization may still be required.

Secondary Research of identifiable private information or identifiable biospecimens may not require informed consent if at least one of the criteria listed below is met. Select at least one of the following criteria and provide a rationale for how the criteria is met:

- Use of publicly available identifiable private information or identifiable biospecimens.

  N/A

- The information and/or biospecimens are recorded by the investigator in such a way that the identity of the subjects cannot be readily ascertained, and the investigator will neither contact the subjects nor re-identify subjects.

  N/A

- The research involves only information collection (i.e., no biospecimen collection) and the analysis of this identifiable health information is regulated by HIPAA.

  Data will be collected on data-only matched controls (receiving usual care subsequent to the regionwide dissemination of HOAP).

**8. HIPAA Privacy Rule Authorization**

a. Written HIPAA Privacy Rule Authorization:
Describe the plan to obtain a signed Privacy Rule Authorization from each subject.

**Phase 2**

A waiver of HIPAA authorization is requested.

b. Waiver of HIPAA Privacy Rule Authorization
If you will not obtain a signed HIPAA Privacy Rule Authorization or if you want to eliminate any required language from the authorization, provide the following rationale and justification.

- Explain why the research could not practicably be conducted without the waiver. Note: research regulations require that justification for a waiver of HIPAA Authorization explain why it is impracticable to perform the *research*, and not just impracticable to obtain HIPAA Authorization. Practicability cannot be determined solely by considerations of convenience/cost/speed.

  Receipt of usual care does not require obtaining HIPAA Privacy Rule Authorization at every step of every encounter. To introduce a consent requirement into the APM encounter would be inappropriate.

- Explain why access to and use of the PHI is necessary for the research.

It is necessary to access PHI to assemble the cohort of study participants and data-only patients. Without access to and use of the necessary PHI the research cannot be conducted. Direct contact with study participants is necessary for usual care as well as for this activity.

- Explain why the use or disclosure of PHI for the research poses no more than minimal risk to the subjects' privacy.

  Use of PHI poses no more than minimal risk as this study will store PHI in password protected or encrypted electronic files and stored on protected KP servers within each region and within KP networks secured by firewalls. Passwords will not be disclosed to any party not involved in the study, including study teams outside of the KP NCAL region. Patient identifiers will not appear in any part of the publications. When it is necessary to share PHI information among study parties within each respective KP region, password-protected/encrypted files will be transmitted through encrypted KP email or secure file transfer. For all these reasons the use and disclosure of PHI poses no more than minimal risk to the subjects' privacy.

- Provide an adequate plan to protect the PHI from improper use or disclosure.

  This study will store PHI in password protected or encrypted electronic files and stored on protected KP servers within KP network secured by firewalls. Passwords will not be disclosed to any party not involved in the study outside of each region. Patient identifiers will not appear in any part of the publications. When it is necessary to share PHI information among study parties within the immediate KP regional team, password-protected/encrypted files will be transmitted through encrypted KP email or secure file transfer. Only designated trained study staff within each regional study team will have access to all data collected as part of this study.

- Provide a plan to destroy identifiers at the earliest opportunity consistent with the purpose of the research.

  Personal identifiers associated with participants will be destroyed as soon as possible after the study has finished and after publication of all manuscripts. Since manuscript submission and revision process often requires re-access of patient information, patient identifiers will be kept until all publications generated from the study have been finished, at which time all patient identifiers will be destroyed at the close of the study.

c. **HIPAA Disclosure Accounting**

The Health Insurance Portability and Accountability Act (HIPAA) Privacy Rule gives patients the right to receive a listing, known as an accounting of disclosure, of their information that is disclosed to others for reasons other than treatment, payment, or health care operations. KP must account for all known disclosures of protected health information for research purposes without the individual's authorization (a waiver or alteration of HIPAA Authorization) both within a KP region (between a Permanente Medical Group, Kaiser Foundation Hospitals, and a Kaiser Foundation Health Plan) and outside of KP.

- What type of PHI is being disclosed?
  ☒ Clinical/Diagnostic
  ☒ Demographics
  ☒ Healthcare

- How many participants are expected to be enrolled in this study at your region?
  ☐ 49 or less individuals

  The investigator should maintain an individual accounting record of all Disclosures made by the research team that are subject to the HIPAA tracking requirements. The investigator must also transmit this tracking information within 20 days of Disclosure to the Regional Compliance Officer. Using: Health Connect Quick Disclosure or Complete the Disclosure of PHI about a single individual for a research purpose Form.

  ☒ 50 or more individuals

  If 50 or more individuals, please provide the information for each entity that sponsored the research where PHI is being disclosed. *Please note: If you are disclosing PHI to more than one entity, the following is needed for each entity.*

  - **Name**
    KPNC Region

  - **Address**
    2000 Broadway, Oakland, CA 94612

  - **Phone Number**
    510-891-3400

If disclosing PHI to more than one entity, please continue entering the required information below:
PHI will not be disclosed outside of the KP NCAL entity.

**9. Study Procedures**

a. Description

Describe and explain the study design, including:

- A detailed chronological description of all research procedures.

**Phase 1:**

Aim 1

Months 1-4: Meetings with stakeholders will be convened by PIs.
Months 4-6: Finalize and disseminate "Hypoglycemia on a Page" (HOAP) (such as dissemination at all regional meetings with APMs, "steal this talk" presentations for PCPs, and electronic posting in the Clinical Library).

Aim 2

Months 2-4: Workflow/clinical pathway will be developed by PIs.

**Phase 2**

Aim 3

Months 5-6:

- Develop procedures and metrics for outcome evaluation
- Identify eligible patients (N=200)(have a high hypoglycemia risk score or a recent ED visit or hospitalization for hypoglycemia); randomly select study participants and data-only patients (matched sample)
- Collect baseline EMR data on  study participants and data-only patients

Months 7-12:

- HC APM conducts outreach to study participants for assessment and evaluation; provides hypoglycemia intervention to study participants.
-

Months 13-21

- Collect outcome data from EMR for study participants and data-only patients.

Months 21-24

- Analyze data and draft manuscript

- Procedures to monitor subjects for safety, including who will review the data and at what frequency for safety issues.

**Phase 2**

APM will routinely monitor study participants for safety and report any concerns to the Investigator.

- Procedures performed to lessen the probability or magnitude of risks.

**Phase 2**

Usual care has no more than minimal risk.

- The source records that will be used to collect information about subjects. (Attach all surveys, scripts, and data collection forms.)

**Phase 2**

Data will be collected from EMR.

- What information and/or biospecimens will be collected including during long-term follow-up.
  *Please note, if biospecimens being collected are being used to develop an investigational in vitro diagnostic device, an IDE for the device may be needed. Please review the IVD Device FAQs in IRBNet for guidance.*

**Phase 2**

Data collected from study participants and data-only patients

- Prescribing orders and pharmacy dispensing of medications including
- Durable medical equipment orders and dispensing of continuous glucose monitor devices and supplies
- Relevant lab results (e.g., HbA1c)
- ED visits or hospital admissions for hypoglycemia
- Hypoglycemia on the problem list
- Visit notes

- The duration of an individual subject's participation in the study.

**Phase 2**

Participation in the study will end within 25 months after enrollment of first participant and no later than 01/31/2026.

- The duration anticipated to enroll all study subjects.

Enrollment is expected to end by 01/31/2026.

- The estimated date for the investigators to complete this study (complete primary analyses) NOTE: It should be clear exactly which procedures will be conducted for the research as opposed to procedures the subjects would undergo (in the exact manner described in the protocol) even if they were not participating in the study.

The study is expected to be completed by 01/31/2026.

- Describe procedures that will be followed when subjects withdraw from the research, including withdrawal from intervention but continued information and/or biospecimen collection.

Participants who become non-adherent to care provided by HC APM will be followed up per the intent to treat analysis plan.

- Describe any anticipated circumstances under which subjects could be withdrawn from the research without their consent.

Discontinuation of Kaiser membership.

- Describe any procedures for orderly termination.

N/A

- If the study involves genetic testing or collection of genetic information, describe this.

N/A

- Clarify whether research involving biospecimens will (if known) or might include whole genome sequencing (i.e., sequencing of a human germline or somatic specimen with the intent to generate the genome or exome sequence of that specimen).

  N/A

- Does the study include basic and clinical research involving recombinant or synthetic nucleic acid molecules, including the creation and use of organisms and viruses containing recombinant or synthetic nucleic acid molecules, and is subject to NIH guidelines (Guidelines: https://osp.od.nih.gov/biotechnology/nih-guidelines/ and FAQs: https://osp.od.nih.gov/biotechnology/faqs-on-ibc-administration/)?

  ☐ Yes
  If yes, your study may require Institutional Biosafety Committee Review. Please contact kpnc.irb@kp.org for guidance.

  ☒ No

  Optional comments:
  N/A

b. Data Analysis
Describe the data analysis plan, including:

- Statistical procedures.

**Phase 2**

Statistical Design: The primary analysis for Aim 3 will be an intent-to-treat analysis using chi square tests and binomial regression. For the primary outcome, differences in the proportion of patients prescribed safer diabetes regimens (i.e., discontinuation of sulfonylureas, mealtime or mixed insulin) will be assessed at 6 months in the treatment vs. control arms. For the secondary outcomes, differences will be assessed in the proportion of patients who had an ED visit or hospitalization for hypoglycemia within 6 months, were prescribed CGM or prescribed glucagon, and were in glycemic control (HbA1c<8%) between the 2 study arms. The investigators will perform exploratory Heterogeneity of Treatment Effect (HTE) analyses for age, race and enrollment criterion (see above) by testing for statistical interactions with the exposure (treatment arm).

- When applicable, the power analysis.

**Phase 2**

Power Calculation: In preparation for this proposal, baseline use of hypoglycemia-prone medications (e.g., sulfonylureas, mealtime, or mixed insulin) was evaluated in 3921 KPNC patients identified as "high risk" by the KPNC hypoglycemia risk tool. Among these patients, 75% were on hypoglycemia-prone treatment. Approximately 60% of the intervention arm and 20% of the usual care arm will be prescribed safer diabetes regimens within 6 months compared

to baseline. The minimum number of subjects needed to detect this 40% absolute difference, assuming 90% power and a 10% attrition rate, is 86 patients (43 per arm). With an enrollment size of 200 (100 per arm), this trial is more than adequately powered to detect the hypothesized difference.

- Any procedures that will be used for quality control of collected data.

Data analysts routinely inspect the data for corrupt fields or extreme outliers.

c. Sharing of Results with Subjects

Describe whether results (study results or individual subject results, such as results of standard or research lab tests and genetic tests) will be shared with subjects or their providers and under which circumstances.

N/A

If the study carries a risk of incidental findings, describe your plan for evaluating these and determining whether and how subjects or their providers will be given this information.
N/A

If laboratory results will be shared with subjects or their healthcare providers, verify that the laboratory conducting the test is Clinical Laboratory Improvement Amendments (CLIA) certified.
N/A

Describe how study participants will be updated on research progress via newsletter, summary, etc. Consider incorporating such updates to study participants as part of ongoing retention efforts.
N./A

## 10. Privacy, Confidentiality and Data Security

Describe the steps that will be taken to protect subjects' privacy during recruitment, consent and study procedures.

Usual care procedures for protecting patient privacy will be used.

Describe the plan for storage of data and/or biospecimens including:

- Who will have access?

The PI, Co-I's and study team will have access to identifiable information from the medical record and other clinical databases. PHI will not be disclosed outside of study team.

- Where the data/materials will be stored and for how long. Indicate if data will be encrypted and password protected and if transportable/removable media will be used. Indicate if data will be stored on non-KP devices or sponsor-provided devices.

Data will be stored in password protected or encrypted electronic files and stored on protected KPNC servers within KP network secured by firewalls. Passwords will not be disclosed to any party

not involved in the study. Patient identifiers will not appear in any part of the publications. When it is necessary to share PHI information among study parties within KPNC, password-protected/encrypted files will be transmitted through encrypted KPNC email or secure file transfer. The information will be stored for the length of the project and destroyed after the final manuscripts are published. Data will be NOT stored on non-KP devices or sponsor-provided devices.

- If applicable, how will data be transmitted? (e.g. Encrypted email, secure file transfer, sponsor provided site)

Data will be transmitted via password-protected/encrypted files through encrypted KPNC email or secure file transfer.

- What identifiers will be included.

  MRNs, dates

- Any other steps that will be taken to ensure security (e.g., training of staff, authorization of access, password protection, encryption, physical security, and separation of identifiers from data and specimens, certificates of confidentiality).

  None, the investigators believe that the plan to protect the data is appropriately mitigated.

- Describe the plan to destroy/archive or retain data at the end of the study. If storing information and/or identifiable biospecimens for future research, complete the next section.

  Personal identifiers associated with participants will be destroyed as soon as possible after the study has finished and after publication of all manuscripts. Since manuscript submission and revision process often requires re-access of patient information, patient identifiers will be kept until all publications generated from the study have been finished, at which time all patient identifiers will be destroyed at the close of the study.

## 11. Information and/or Biospecimen Banking for Future Research

If you are creating a repository, please submit a separate protocol.

Indicate if biospecimens may be used for future research and whether that may include genetic research.

N/A

State if information or biospecimens will be sent to a separate repository. If data or specimens will be banked in a repository for future use as part of this protocol submission address the following questions:

- What will be banked and what identifiers will be associated with the information or biospecimens?

N/A

- Where and how will the information or specimens be stored?

  N/A

- For what purpose will the information or specimens be used? Include a general description of the types of research that may be conducted with the identifiable private information/biospecimens (e.g. research on cancer).

  N/A

- How will the information or specimens be accessed, and who will have access?

  N/A

- Describe the procedures to release information or specimens, including the process to request a release, approvals required for release, who can obtain information or specimens, and the information to be provided with specimens.

  N/A

- How long will identifiable private information/biospecimens be stored?

  N/A

**12. Collection of data from subjects electronically**

If you will collect any data from participants electronically (including email, website, etc.), explain:

- Does the study involve a Mobile Device or Application? If yes, please indicate the source/manufacturer/developer of the product:

  No.

- How and what data will be collected. Indicate if PHI is collected electronically (including via an app) and if there is any interface with the subject's personal accounts (for example: if subjects need to create an account to use the device, or if the device interfaces with an external source of data, or medical record).

  N/A

- How the information will be secured/transmitted/stored (encryption, password protection, etc.; may require consultation with IT department). Include a data flow diagram if data will flow through multiple parties (such as a hosting provider or coordinating center).

  N/A

- Any risks to the participants' privacy posed by using these methods (describe in consent, as applicable).

  N/A

- Describe if email containing PHI will be used to communicate with participants? (Per KP policy, securing messaging must be used for all communications containing PHI.)

  N/A

- How you will verify the participant's identity.

  N/A

**13. Disclosure of PHI to a collaborator**
If any data will be sent outside of this site, list each recipient (may list by role or category if the information is the same for several different entities). For each recipient, describe:

- The name and location of the individual/entity receiving the information.

  N/A

- What information and/or biospecimens will be sent.
  N/A

- Whether the information will be fully identifiable (PHI, if health information), a Limited Data Set, de-identified, or aggregate.

  N/A

- How the data/materials will be transferred securely (for instance, Secure File Transfer). Indicate if hard copy PHI will be stored/sent to a collaborator.

  N/A

- When applicable, clarify whether there are written assurances from collaborators that PHI will not be reused or re-disclosed to any other entity. Describe assurances that PHI will be stored securely. Describe mechanisms (e.g., intrusion detection software or regular electronic system activity audits or monitoring) for determining if KP PHI has been inappropriately or illegally accessed, used, disclosed, or modified.

  N/A

**14. Provisions to Monitor Data to Ensure the Safety of Subjects**
This is required when research involves more than Minimal Risk to subjects.

The plan might include establishing a data monitoring committee and a plan for reporting data monitoring committee findings to the IRB and the sponsor. Describe:

- Who will monitor the study data for safety?

  N/A. This study involves no more than minimal risk to subjects.

- The plan to periodically evaluate the data collected regarding both harms and benefits to determine whether subjects remain safe.

  N/A

- What data are reviewed, including safety data, untoward events, and efficacy data.

  N/A

- How the safety information will be collected (e.g., with case report forms, at study visits, by telephone calls with participants).

  N/A

- The frequency of data collection, including when safety data collection starts.

  N/A

- The frequency or periodicity of review of cumulative data.

  N/A

- Criteria for taking action on monitoring findings (for instance, stopping rules, immediate suspension, reporting, protocol changes, changes to monitoring frequency or plan).

  N/A

- For studies monitored by a DSMB/C, describe the committee membership and structure, meeting format, and quorum requirements. Upload the board/committee charter, if one exists.

  N/A

**15. Risks and Benefits**

a. Risks to Subjects

List the reasonably foreseeable risks, discomforts, hazards, or inconveniences to the subjects related the subjects' participation in the research. Consider physical, psychological, social, legal, and economic risks.

No foreseeable risks, discomforts, hazards, or inconveniences to participants are anticipated.

Describe the probability, magnitude, duration, and reversibility of the risks.
N/A

If applicable, indicate which procedures may have risks to the subjects that are currently unforeseeable.

N/A

If applicable, indicate which procedures may have risks to an embryo or fetus should the subject be or become pregnant.

N/A

If applicable, describe risks to others who are not subjects and risks to Kaiser Permanente

N/A

b. Potential Benefits to Subjects
Describe the potential benefits that individual subjects may experience from taking part in the research. Include as may be useful for the IRB's consideration, the probability, magnitude, and duration of the potential benefits. Indicate if there is no direct benefit. Do not include benefits to society or others.

The study is intended to reduce risk of hypoglycemia and improve patient safety. Participants may experience these benefits.

c. Risks to KP
Is there anything about the nature of this study which, if revealed to the public, could put KP at risk or competitive disadvantage? ☐ **Yes** ☒ **No**
If "yes" describe in detail.

**16. Economic Burden to Subjects**
Describe any costs that subjects may be responsible for because of participation in the research study (for example, co-pays; paying for treatment, therapies, or other interventions, or the delivery of these) and how you will inform participants of these costs prior to their enrollment in this study.

None

**17. Compensation to Participants**
Describe any compensation provided to participants, for example, for time inconvenience, discomfort, travel, or in the event of research related injury. If applicable, describe how you will inform participants of this prior to their enrollment in the study, including if payment will be prorated if the subject withdraws early from the study.

NOTE: payment may not be withheld as an incentive for participants to complete any portion of the study.

None.

**18. Resources Available**
Describe any special resources or expertise required to conduct the study.

Click or tap here to enter text.

**19. Principal Investigator**

a. Has the Principal Investigator (PI) previously been approved as PI for a study within KPNC? ☒ Yes ☐ No

b. Has the PI been audited/assessed within the past three years? ☐ Yes ☒ No
If Yes, check all that apply and provide the outcome/findings:
☐ FDA ☐ RQCC ☐ CTP ☐ CCRU
Outcome/Findings: Click or tap here to enter text.

c. Is the PI currently under a Corrective And Preventative Action (CAPA) plan?
☐ Yes ☒ No
If Yes, provide a brief description: Click or tap here to enter text.

**20. Required Approvals**

a. Describe any approvals that will be obtained prior to commencing the research. (e.g., school, external site. funding agency, or other KP departments). Be sure to list each KP site (for example, KP San Francisco Hospital, Division of Research, KP Oakland Reg – 1800 Harrison, etc.)

Division of Research

b. **Facility-Based Research:** KPNC IRB requires that the Principal Investigator (PI) obtain approval signatures at **each KPNC facility** where research activity will occur. These may include local research chair, Physician-in-Chief, departmental chair, Area Manager, information technology review (as appropriate), and Division of Research scientific review (student research).

List each KP facility that research activity will occur and specify whose signature has been/will be obtained.

Name of Facility: SSF

Local Research Chair (LRC): Robert Li

Chief of Service: Joanie Loh, MD

Physician-in-chief (PIC): John Skerry, MD

Area Managers (if applicable): Sheila Gilson

c. **Non-Facility-Based Research** (ex: Northern California Regional and Program Office): For non-facility-based research, the PI's supervisor and department head are required in lieu of approvals from the Chief of Service, Physician-in-chief, and Area Manager. The Central Research Committee Chairperson serves as the LRC. As such, list each KP non-facility that research activity will occur and confirm whose signature has been/will be obtained.

PI's Supervisor: Tracy Lieu, MD (Richard Grant)

Department Head: ?

Central Research Committee Chairperson: Michael Silverberg, MD

d. **Sites with no Local Research Chair:** For sites with no Local Research Chair, the Central Research Committee Chairperson serves as the Local Research Chair:

Site: N/A

Central Research Committee Chairperson: N/A

**21. Drugs or Devices**

NOTE: see the ICH-GCP guidance for a summary of investigator and sponsor responsibilities in clinical trials.

List all drugs and devices used in the research and the purpose of their use, and their regulatory approval status.

N/A

a. Drug Studies

If the research involves drugs and is investigator-initiated, indicate whether there is any possibility that the results will be reported to FDA (e.g. as part of a new drug application [NDA]).

N/A

If the drug is investigational (has an IND), confirm that you will comply with all applicable FDA requirements for investigators.

N/A

Confirm that you will follow applicable KP pharmacy policies and procedures.

N/A

Describe your plan for drug storage, handling, and accountability, including distribution, return, and destruction of the drug(s).

N/A

b. Device Studies:

If this is a device study and you think the device is Non-Significant Risk, include justification here or upload it as a separate document along with any available device information (instructions for use, etc.).

N/A

If the research involves devices and is investigator-initiated, indicate whether there is any possibility that the results will be reported to FDA (e.g. as part of a premarket approval application [PMA]).

N/A

If the device has an IDE or a claim of abbreviated IDE (Non-Significant Risk device), confirm that you will comply with all applicable FDA requirements for investigators.

N/A

Describe the device, the manufacturing process, and the device labeling, including safety instructions or warnings. If available, this may be addressed in separately uploaded device information (such as instructions for use).

N/A

Describe device storage, handling, and accountability, including how access to the device will be limited to appropriate personnel and how you will ensure the device will be used only for appropriate study subjects.

N/A

**22. Multi-Site Research**

a. If this is a multi-site study and you are the lead investigator or this site will be the coordinating center for any activity, describe the processes to ensure communication among sites, such as:

- All sites have the most current version of the protocol, consent document, and HIPAA authorization.

  *N/A*

- All required approvals have been obtained at each site (including approval by the site's IRB of record).

  *N/A*

- All modifications have been communicated to sites, and approved (including approval by the site's IRB of record) before the modification is implemented.

  *N/A*

- All engaged participating sites will safeguard data as required by local information security policies.

  *N/A*

- All local site investigators conduct the study appropriately.

*N/A*

b. Describe the method for communicating to engaged participating sites the following:

- Problems.

  N/A

- Interim results.

  N/A

- The closure of a study.

  N/A

c. Describe any special resources or expertise required to conduct the study.

*N/A*

**23. Community-Based Participatory Research**

Describe involvement of the community in the design and conduct of the research.

N/A

Describe your plan for ensuring that community research partners are appropriately trained in human subjects' protection.

N/A

NOTE: "Community-based Participatory Research" is a collaborative approach to research that equitably involves all partners in the research process and recognizes the unique strengths that each brings. Community-based Participatory Research begins with a research topic of importance to the community, has the aim of combining knowledge with action and achieving social change to improve health outcomes and eliminate health disparities.
