# Supplementary material for: Pharmacist Intervention for Safer Prescribing in Patients With Type 2 Diabetes at High Risk: A Randomized Clinical Trial
Source: JAMA Netw Open. 2026 Feb 18;9(2):e2559946. doi: 10.1001/jamanetworkopen.2025.59946 (PMC12917679; doi:10.1001/jamanetworkopen.2025.59946)
Supplement: Supplement 2. — Data Sharing Statement [file jamanetwopen-e2559946-s002.pdf]

Data Sharing Statement

Gilliam. Pharmacist Intervention for Safer Medication Prescribing in Patients With Type 2 Diabetes at High Risk. *JAMA Netw Open*. Published February 18, 2026. doi:10.1001/jamanetworkopen.2025.59946

Data
Additional Information: The trial was registered in the government list clinical trials (clinicaltrials.gov NCT06746714). https://clinicaltrials.gov/study/NCT06746714?term=NCT06746714&rank=1
Data available: No

Additional Information
Explanation for why data not available: Data cannot be made available due to IRB restrictions and health system policies.
